# Treatments for NAFLD: State of Art

**DOI:** 10.3390/ijms22052350

**Published:** 2021-02-26

**Authors:** Alessandro Mantovani, Andrea Dalbeni

**Affiliations:** 1 Section of Endocrinology, Diabetes and Metabolism, University and Azienda Ospedaliera Universitaria Integrata of Verona, 37126 Verona, Italy; 2 Section of General Medicine, Hypertension and Liver Unit, University and Azienda Ospedaliera Universitaria Integrata of Verona, 37134 Verona, Italy; andrea.dalbeni@aovr.veneto.it

**Keywords:** non-alcoholic fatty liver disease, NAFLD, non-alcoholic steatohepatitis, metabolic associated fatty liver disease, MAFLD

## Abstract

Non-alcoholic fatty liver disease (NAFLD) is to date the most common chronic liver disease in clinical practice and, consequently, a major health problem worldwide. It affects approximately 30% of adults in the general population and up to 70% of patients with type 2 diabetes (T2DM). Despite the current knowledge of the epidemiology, pathogenesis, and natural history of NAFLD, no specific pharmacological therapies are until now approved for this disease and, consequently, general strategies have been proposed to manage it. They include: (a) lifestyle change in order to promote weight loss by diet and physical activity, (b) control of the main cardiometabolic risk factors, (c) correction of all modifiable risk factors leading the development and progression of advanced forms of NAFLD, and (d) prevention of hepatic and extra-hepatic complications. In the last decade, several potential agents have been widely investigated for the treatment of NAFLD and its advanced forms—shedding some light but casting a few shadows. They include some glucose-lowering drugs (such as pioglitazone, glucagon-like peptide-1 (GLP-1) receptor agonists, sodium-glucose co-transporter-2 (SGLT-2) inhibitors), antioxidants (such as vitamin E), statins or other lipid lowering agents, bile and non-bile acid farnesoid X activated receptor (FXR) agonists, and others. This narrative review discusses in detail the different available approaches with the potential to prevent and treat NAFLD and its advanced forms.

## 1. Introduction

Non-alcoholic fatty liver disease (NAFLD) is the hepatic manifestation of metabolic syndrome and includes a spectrum of progressive conditions, such as simple steatosis, steatohepatitis (NASH), fibrosis (with various grades of severity), and cirrhosis [1,2]. To date, NAFLD is the most common chronic liver disease seen in clinical practice, mostly in high-income countries. In fact, NAFLD affects approximately 30% of adults in the general population [3] and up to 70% of patients with type 2 diabetes mellitus (T2DM) [4,5].

Though the physiopathology of NAFLD is complex, a large body of evidence now suggests that NAFLD, insulin resistance, obesity, and T2DM frequently coexist and play a role in the development of adverse hepatic and extra-hepatic clinical outcomes, such as cardiovascular diseases (that are the leading causes of death in NAFLD patients) [5,6,7,8,9,10]. In 2020, some experts in the field have proposed a change of the terminology from NAFLD to metabolic associated fatty liver disease (MAFLD) [11,12]. The diagnosis of MAFLD can be performed by the presence of hepatic steatosis (as detected by specific serum biomarker scores, imaging techniques, or histology) and at least one of the following criteria: (a) overweight or obesity, (b) T2DM, and (c) metabolic dysregulation (at least two factors among increased waist circumference, hypertension, hypertriglyceridemia, low serum HDL-cholesterol levels, impaired fasting plasma glucose, insulin resistance (assessed by homeostatic model assessment of insulin resistance), or subclinical inflammation (evaluated by high-sensitivity C-reactive protein levels)) [11,12]. However, seeing that at the time of writing it is still debated which term should be used in literature [13,14], we have considered it appropriate to maintain the NAFLD term in this narrative review.

**Figure 1:**
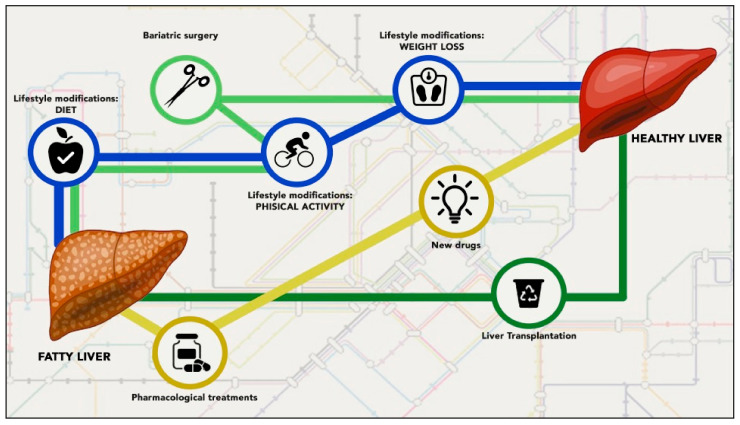
Current proposed treatments for non-alcoholic fatty liver disease (NAFLD) and its advanced forms. Although specific therapies for NAFLD are not approved yet, general strategies have been proposed to manage it, including: (a) lifestyle change (blue line), (b) glucose-lowering agents (including pioglitazone), or antioxidants (such as vitamin E), or other promising drugs (yellow line), (c) bariatric surgery in patients with severe obesity (i.e., BMI > 35 kg/m^2^) (light green line), and (d) liver transplantation in selected cases (dark green line).

Despite our understanding of the mechanisms involved in the development and progression of NAFLD, at present there are no approved pharmacological treatments for NAFLD and its advanced forms in patients with and without T2DM [1,2,15,16]. The management of NAFLD is based essentially on four goals: (a) lifestyle change in order to promote weight loss; (b) control of the cardiometabolic risk factors; (c) correction of all modifiable risk factors leading to the development and progression of advanced forms of NAFLD; and (d) prevention of hepatic and extra-hepatic complications [1,2,15,16,17]. Hypothetically, an optimal therapy for NAFLD should reduce hepatic steatosis, inflammation, and even fibrosis in order to prevent serious liver-related complications. In addition, it should also improve the metabolic background with the aim to prevent T2DM, cardiovascular diseases, and extra-hepatic cancers. It is reasonable to suppose that a single agent (or approach) is unable to achieve these goals. Hence, several beneficial therapeutic approaches exist (as summarized in Figure 1) and are being tested in several studies [16].

The aim of this narrative review is to summarize the current proposed treatments for NAFLD in patients with and without T2DM and to discuss the potential future treatments, current trials, and unanswered questions.

### 1.1. Lifestyle Modifications

A large body of evidence strongly supports the role of lifestyle modification as the primary approach for the management of NAFLD and its advanced forms [1,2,15,16,18]. Weight loss is able to regress liver disease, along with the reduction of cardiovascular diseases and T2DM risk [18]. A weight reduction of ≥10% can produce NASH resolution, as well as fibrosis improvement by at least one stage [18]. Modest weight loss (from 5% to 10%) can exert benefits on various components of the NAFLD activity score (NAS) [18]. For such reasons, the EASL-EASO-EASD and American Association for the Study of Liver Diseases (AASLD) practice guidelines for the management of NAFLD indicate that, in overweight/obese NAFLD patients, a 5–10% weight loss is the main goal of most lifestyle interventions [1,2]. Similar recommendations were also provided by National Institute for Health and Care Excellence (NICE) guidelines [15] (see Table 1). Some evidence also documents that non-obese NAFLD patients can achieve NAFLD remission with a modest weight loss of 3–10% and that they are more likely, compared with obese NAFLD patients, to maintain weight reduction and normal liver enzymes over time [19].

The effect of weight loss on histological improvement appears to be in relation with the degree of weight reduction, rather than the method used to reach it [18]. Indeed, lifestyle intervention, including hypocaloric diet and physical exercise, weight loss-induced by drugs (e.g., orlistat), or weight loss after bariatric surgery, seems to have a similar favorable effect on NASH resolution and fibrosis regression [18]. All patients with NAFLD, regardless of the presence of T2DM, should avoid alcohol consumption (even moderate) and, when possible, the use of hepatotoxic drugs [1,2]. Clinicians should also recommend avoiding cigarette smoking [1,2,15,20] and fructose-containing beverages and foods [1,2,15,21].

Several different diets have been tested in patients with NAFLD [18,22]. Observational studies and small randomized controlled trials (RCTs) have consistently documented that the Mediterranean diet has favorable effects in patients with NAFLD and, in addition, it is able to reduce liver fat, as well as improve metabolic profile, regardless of weight loss [22,23,24,25]. For such reasons, at present, the Mediterranean diet is the most recommended dietary pattern in NAFLD patients by most guidelines [1,2,22]. In brief, the Mediterranean diet is characterized by a high intake of vegetables, legumes, whole grains, olive oil (as the main source of added fat), fish, seafood, nuts, fruits, as well as a low intake of red meat, processed meats, and sweets [18,22,26]. Compared with low fat diets that usually contain up to 30% fat, in the Mediterranean diet 40% of the calories comes from fats, especially monounsaturated fat and omega-3 polyunsaturated fatty acids [22,26]. In addition, it is clearly documented that the Mediterranean diet is able to reduce the risk of cardiovascular disease [27,28] and T2DM [29], i.e., two conditions commonly observed in NAFLD patients [5,6,30]. By contrast, the consumption of components characterizing a Western dietary pattern, including soft drinks, fructose, processed meat, and saturated fatty acids, has been shown to have detrimental effects on the development and progression of NAFLD [18,22,25]. In a small randomized, cross-over trial enrolling 12 non-diabetic patients with biopsy-proven NAFLD, randomly assigned to Mediterranean diet or low fat-high carbohydrate diet over a follow-up of 6 weeks, Ryan et al. reported that the Mediterranean diet reduced liver steatosis (−39% vs. −7%, as measured by magnetic resonance spectroscopy, *p* < 0.05) and improved insulin sensitivity [31]. Similar findings were also reported in patients with T2DM [32]. In a small randomized, parallel-group design trial enrolling 45 T2DM patients randomly assigned to two 8-week isocaloric diets (a high-carbohydrate/fiber diet or a high-monounsaturated fatty acid diet), Bozzetto et al. reported that liver fat content, as measured by magnetic resonance spectroscopy, decreased more in the high-monounsaturated fatty acid group, as compared with the high-carbohydrate/fiber group (−29% vs. −4%, *p* < 0.05, respectively) [32]. Recently, in a randomized, parallel-group design trial of 51 obese patients with NAFLD who were randomly assigned to a 12-week single-blinded dietary intervention (i.e., ad libitum isocaloric Mediterranean diet or low-fat diet), Properzi et al. documented that hepatic steatosis (evaluated by magnetic resonance spectroscopy) decreased significantly in both groups, with no difference in terms of mean percentage reductions in liver fat content [33]. Potential molecular mechanisms for the favorable effects of the Mediterranean diet include the presence of polyphenols, carotenoids, oleic acid, polyunsaturated fatty acids (PUFAs), and fiber [22,24]. These components might favorably influence some pathways involving the intestine, adipose tissue, and liver, thereby mediating favorable effects in NAFLD [24,34,35]. While there are strong data supporting the role of dietary changes as primary therapy in NAFLD treatment, a potential clinical problem is the adherence of the patient to beneficial changes in the diet [34]. In this context, it is also important to note that the Mediterranean diet may not be practical in each country or in every population [22]. Hence, some authors have proposed a “wise choices” attitude, by which a patient makes the best nutritional choices, trying to keep at least some of the principles of the Mediterranean diet [22]. Given that each component of the Mediterranean diet has a beneficial effect on NAFLD, this may be an acceptable compromise in clinical practice [22,34].

Different forms of physical exercise (e.g., aerobic exercise, resistance exercise, or high intensity intermittent exercise) seem to have similar effects on liver fat content [16,18,36,37]. However, in the study by Oh et al., resistance training, high-intensity interval aerobic training, and moderate-intensity continuous aerobic training were equally effective in reducing hepatic fat content, but only high-intensity interval aerobic training was effective in improving hepatic stiffness and restoring Kupffer cell function [38]. Notably, these benefits appeared to be independent of weight loss and visceral fat reduction [38]. 

A 2017 systematic review of 24 studies reported that exercise, irrespective of weight loss, produced a 20–30% relative reduction in intrahepatic triglyceride content, as measured by proton magnetic resonance spectroscopy [39]. However, it is important to highlight that, in the aforementioned meta-analysis, most studies had a small sample size and were heterogeneous. Recently, in an RCT of 220 obese NAFLD patients who were randomly assigned to vigorous/moderate exercise, moderate exercise, or no exercise (controls), Zhang et al. showed that intrahepatic triglyceride content (on proton magnetic resonance spectroscopy) was significantly reduced in the two exercise groups, as compared with the control group, over the 12-month active intervention [40]. However, if patients do not continue to exercise, the benefits are then lost [18]. Interestingly, genetic background (e.g., *PNPLA3* rs738409 variant) in NAFLD patients might influence their response to physical activity. For instance, accumulating evidence now shows that NAFLD patients with rs738409 G/G genotype respond better to lifestyle intervention, when compared to those with rs738409 G/G CC or C/G [18,41]. In addition, NASH resolution and fibrosis regression rate after lifestyle intervention seem to be reduced also in other specific patients, such as older people, T2DM patients, and those with higher histological activity in liver biopsy (NAS > 5) [18]. The mechanisms underpinning the change in liver fat content due to exercise display changes in energy balance, insulin sensitivity, and circulatory lipids [16,18,42,43]. Exercise improves hepatic and systemic insulin sensitivity, thereby producing an improvement in insulin action and reducing de novo lipogenesis [18,42,43]. Exercise also has direct effects on glycemic control and lipid flux, with an increase in very-low-density lipoproteins (VLDL) clearance, thereby promoting reduction of liver fat content [18,42,43]. In addition, an important reduction in visceral adipose tissue also occurs with exercise [18,42,43]. Visceral fat directly correlates with liver inflammation and fibrosis, independent of insulin resistance and hepatic steatosis [18,42,43]. Lastly, given that patients with NAFLD are at higher risk of developing cardiovascular disease than those without, it is important to remember that the beneficial effects of exercise are extended to the entire cardiovascular system [18,44].

### 1.2. Bariatric Surgery

Bariatric surgery consists of surgical procedures causing weight loss by restricting the amount of food the stomach can hold and/or by promoting malabsorption of nutrients. Worldwide, the most common bariatric surgery procedures are laparoscopic sleeve gastrectomy, laparoscopic Roux-en-Y gastric bypass, laparoscopic adjustable gastric banding, and duodenal switch. Along with a reduction of body weight, bariatric surgery is able to improve insulin resistance, obesity, T2DM, hypertension, dyslipidemia, and obstructive sleep apnea [45]. Importantly, bariatric surgery can markedly improve all histological features of NAFLD, including fibrosis [1,2,45]. In a 2019 systematic review and meta-analysis of 32 cohort studies comprising 3093 biopsy specimens, Lee et al. showed that bariatric surgery resulted in a biopsy-confirmed resolution of steatosis in 66% of patients (95% confidence interval 56–75%), inflammation in 50% (95% confidence interval 35–64%), ballooning degeneration in 76% (95% confidence interval 64–86%), and fibrosis in 40% (95% confidence interval 29–51%) (46). However, these effects seem to not be consistent, as a subset of patients may progress to advanced forms of NAFLD after surgery, whereas others may develop de novo NAFLD. In the aforementioned meta-analysis, for instance, the authors found that NAFLD bariatric surgery resulted in new or worsening features of NAFLD, including fibrosis, in 12% of patients (95% confidence interval 5–20%) [46].

The potential liver-related benefits derived from bariatric surgery may extend beyond weight loss [47]. Indeed, bariatric surgery can increase circulating levels of glucagon-like peptide-1 (GLP-1), which, in turn, decreases appetite, slows gastric emptying, and improves insulin sensitivity [47]. In addition, GLP-1 modulates bile acid signaling via the farnesoid X receptor (FXR), which can alter the gut microbiome and promote NAFLD [48,49]. With these premises, current guidelines indicate that bariatric surgery can be a potential option in patients with T2DM or in those with severe obesity (i.e., BMI >35 kg/m^2^) [1,2]. Bariatric surgery should also be considered as an alternative option in patients with a BMI between 30 and 35 kg/m^2^ when T2DM is not adequately controlled by optimal medical regimen, mainly in the presence of cardiovascular disease (CVD) risk factors [1,2]. While bariatric surgery is effective, there are important limitations, including complications (e.g., bleeding, infection, adverse reactions to anesthesia, blood clots, leaks in the gastrointestinal system, bowel obstruction, dumping syndrome, flushing, nausea, vomiting, diarrhea, gallstones, hernias, malnutrition, acid reflux, and even death), patient acceptability, service availability and costs [45]. For these reasons, the potential side effects and long-term consequences of bariatric surgery should be carefully considered [1,2]. Although the beneficial effects of bariatric surgery are clinically relevant, long-term prospective studies (including RCTs) are required to establish whether remission of NAFLD is permanent, as well as to determine the exact frequency of the recurrence of NAFLD in specific patients. In addition, no consistent evidence regarding the comparative effects of different bariatric procedures on liver fat content is available to date.

### 1.3. Liver Transplantation

NASH-associated cirrhosis is to date among the top three indications for liver transplantation in most high-income countries, with the alarming trajectory to become the most common [1,2]. Outcomes at 1, 3, and 5 years for patients undergoing a liver transplant for NASH-associated cirrhosis are substantially similar to those for other indications [50,51,52]. However, the overall mortality in patients with NASH-associated cirrhosis undergoing liver transplant seems to be more frequently associated with the age of the recipient (i.e., >60 years), presence of obesity (i.e., BMI ≥ 30 kg/m^2^), T2DM, or post-transplant MetS [50,52,53,54]. Recurrence of NAFLD is also common after liver transplantation, ranging from 20% to 40% of cases, depending by the techniques used for the diagnosis [50,52,53,54]. Risk factors associated with NAFLD recurrence include: (a) post-transplant weight gain, (b) steroid use, (c) presence of MetS, and (d) *PNPLA3* rs738409 in the recipient [50,52,53,54]. In addition, approximately 30% of patients transplanted for reasons different from NASH-associated cirrhosis can develop de novo NAFLD, usually within 3 years of the liver transplantation [50,52,53,54]. Specifically, T2DM, obesity, arterial hypertension, and liver graft steatosis are the most important risk factors for de novo NAFLD [50,52,53,54]. Consequently, based on these data, close management of the components of MetS is relevant for long-term survival in these patients [50,52,53,54]. In addition, patients with NASH-associated cirrhosis tend to have poor performance status, which has been associated with decreased graft survival and overall patient 5-year survival rates [50,52,53,54].

There are some challenges faced by patients with NASH-associated cirrhosis undergoing liver transplantation (54). First, the increasing prevalence of obesity and T2DM has significantly contributed to an increase in the presence of hepatic steatosis in potential donors, thereby reducing the number of transplantable livers [54]. In fact, donor livers with >60% steatosis are to date judged non-transplantable, whereas those with <30% are considered useable [54]. Livers with 30–60% steatosis may be used for specific patients, although they tend to be associated with poor results [54]. Second, given that NAFLD is the liver manifestation of MetS, patients with NASH-associated cirrhosis frequently have many important comorbidities, such as obesity, cardiovascular disease, T2DM, and chronic kidney disease [54]. In this regard, AASLD practice guidelines consider severe obesity (i.e., BMI ≥ 40 kg/m^2^) as a relative contraindication for liver transplantation [2], whereas EASL-EASD-EASO practice guidelines state that a multidisciplinary team should always evaluate patients with a BMI > 35 kg/m^2^ before being included on the waiting list [1]. In addition, AASLD practice guidelines also recommend that patients with NASH-associated cirrhosis should be carefully evaluated for identifying CVD during the transplant evaluation process [2]. Third, patients with NASH-associated cirrhosis who are on the waiting list for liver transplantation may compete for liver allograft allocations, due to lower Model for End-Stage Liver Disease (MELD) scores when compared with other etiologies of chronic liver disease [55]. In fact, patients with end-stage liver disease due to NASH on the waiting list for liver transplantation may have better liver functioning and, consequently, lower MELD scores when compared with other etiologies of liver cirrhosis.

Additional research is, however, needed for improving the health care of this increasing patient population.

### 1.4. Pharmacological Approach

Although at present there are no specific agents approved for the treatment of NAFLD and its advanced forms, several potential agents have been widely investigated in the last decades, including glucose-lowering drugs (such as metformin, pioglitazone, GLP-1 receptor agonists, and sodium-glucose co-transporter-2 (SGLT-2) inhibitors), statins and other lipid lowering therapies, anti-hypertensive drugs, and other possible agents, such as obeticholic acid. A summary of principal agents and their characteristics for treating NAFLD and its advanced forms is reported in Table 2.

### 1.5. Metformin

Metformin is a biguanide that is broadly recommended as the initial pharmacotherapy in most patients with T2DM at the time of diagnosis [84,85]. Metformin is considered safe and effective (with a reduction in HbA1c levels ranging from 0.5% to 1%) in T2DM patients with no contraindications (including chronic kidney disease stage 4 or 5, advanced heart failure, advanced pulmonary disease, or history of lactic acidosis) [84]. Experimentally, metformin reduces blood glucose levels by mechanisms involving an AMP-activated protein kinase (AMPK)-dependent improvement of hepatic glucose metabolism and increased glucose uptake into muscle cells [84,86]. Other mechanisms involving alterations in cellular energy charge, fructose-1,6-bisphosphatase 1, and modulation of the cellular redox state through direct inhibition of mitochondrial glycerol-3-phosphate dehydrogenase have been proposed as potential factors of inhibition of gluconeogenesis by this glucose-lowering agent [87]. Metformin can also reduce the risk for cardiovascular events and death in T2DM patients who are overweight or obese [84,85]. However, in the RCTs involving adults with biopsy-proven NAFLD, despite its beneficial effects on liver enzymes and HbA1c levels, metformin showed small beneficial effects on liver steatosis or inflammation but no effects on the resolution of NASH and liver fibrosis [56]. Moreover, in the Treatment of NAFLD in Children (TONIC) trial involving obese children/adolescents with biopsy-proven NASH, metformin completely failed to show any beneficial effect on various features of liver histology [57]. Although metformin is generally well tolerated, gastrointestinal symptoms (such as diarrhea, bloating, or abdominal pain) can occur in a subset of T2DM patients [84]. Long-term use of metformin has also been associated with vitamin B12 deficiency [84]. At present, the EASL-EASO-EASD and AASLD practice guidelines for the management of NAFLD do not support the use of metformin for the treatment of NAFLD [1,2]. NICE guidelines are in line with these recommendations [15]. However, the possible advantages of metformin in chronic liver disease seem to be restricted to its potential effect in reducing the risk of cirrhosis and HCC [88,89,90,91,92]. Indeed, several case-control and cohort studies, as well as some meta-analyses, have documented an independent association between metformin and reduction in HCC incidence among T2DM patients with chronic liver disease [88,89,90,91,92]. For instance, in a retrospective cohort study involving 191 T2DM patients with biopsy-proven NASH and bridging fibrosis or compensated cirrhosis, Vilar-Gomez et al. documented that long-term metformin use was independently associated with reduced risk of all-cause mortality and HCC incidence over a mean follow-up of 7 years [89]. In a 2020 meta-analysis of six retrospective cohort studies (four studies with curative treatment for HCC, for a total of 618 patients with metformin and 532 patients with other anti-hyperglycemic agents, and two studies with non-curative treatment for HCC, for a total of 92 patients with metformin and 57 patients with other glucose-lowering agents), Zhou et al. reported that treatment with metformin was associated with significantly longer overall survival and recurrence-free survival rates in HCC patients, when compared to other glucose-lowering agents [90]. Experimental evidence suggests that metformin may inhibit cancer invasion and metastasis by AMPK (AMP-activated protein kinase) signaling pathway, EMT (epithelial–mesenchymal transition) signaling pathway, and specific epigenetic modifications, thereby improving the prognosis of patients with cancer [93]. However, although this evidence indicates that metformin may be associated with a reduction of cancer prevalence and incidence in patients with T2DM, specific randomized controlled trials are needed to corroborate the aforementioned findings provided by case-control and cohort studies.

### 1.6. PPAR-Gamma Agonists

Rosiglitazone and pioglitazone (which is to date the only glitazone agent available on the market) are two selective ligands of the peroxisome proliferator-activated receptor (PPAR)-γ [84,85]. Briefly, peroxisome proliferator-activated receptors (PPARs), constituted by three different isotypes (i.e., α, β/δ and γ), are nuclear regulatory factors modulating key elements of glucose and fat metabolism [58]. In addition, they can also regulate inflammatory cell activation and fibrotic processes [58]. In this way, binding PPAR-γ, glitazones modulate insulin action, glucose, and lipid metabolism, as well as inflammation and adipose tissue biology [58,84,85]. The PPAR-γ has three isoforms [58,84,85]. Among the three known PPAR-γ isoforms, the PPAR-γ receptor-2 isoform is particularly expressed in adipose tissue, playing a key role in the redistribution of intra-abdominal and subcutaneous adipose tissue by promoting accumulation of triglyceride in peripheral adipose tissue depots [58,84,85]. PPAR-γ is also expressed in Kupffer cells that are involved in the fibrosis processes [58,84,85]. In the context of cirrhosis, PPAR-γ can reduce portal pressure, inflammation, angiogenesis, and portosystemic shunts [58]. Given these pre-clinical data [58,84,85], several studies [59,60,61,62,63] have been performed to test the potential beneficial effects of glitazones in NAFLD/NASH patients. In this regard, for instance, a recent systematic review shows that the use of pioglitazone in patients with biopsy-confirmed NASH can have important benefits on liver function, liver fat content, and resolution of NASH in patients with and without T2DM [56]. Conversely, as compared to its beneficial effects on NASH, the effect of pioglitazone on liver fibrosis seems to be relatively modest [56]. However, in a placebo-controlled RCT of 101 adults with biopsy-proven NASH and T2DM who were randomly assigned to pioglitazone (45 mg once daily) or placebo for 18 months, Cusi et al. documented that approximately 60% of patients in the pioglitazone group achieved the primary outcome, defined as a reduction of at least 2 points in the NAFLD activity score without worsening of fibrosis, and nearly 50% of them had full resolution of NASH [61]. Long-term pioglitazone treatment improved individual histologic scores of NASH, including fibrosis score [61]. In a meta-analysis of eight RCTs enrolling nearly 500 adults with biopsy-confirmed NASH followed up to 24 months, Musso et al. documented that pioglitazone improved advanced fibrosis in NASH patients, regardless of T2DM [64]. Data for rosiglitazone are, instead, more limited. The 1-year FLIRT (Fatty Liver Improvement with Rosiglitazone Therapy) trial documented that steatosis significantly improved in patients receiving rosiglitazone, as compared with those receiving placebo (47% vs. 16%, respectively, *p* < 0.05). By contrast, no significant changes in liver fibrosis were observed [63]. Studies on the rosiglitazone were, however, stopped for an increased cardiovascular risk [85]. Based on these data, the EASL-EASO-EASD, AASLD, and NICE practice guidelines for the management of NAFLD recommend the use of pioglitazone in patients with NASH [1,2,15]. However, pioglitazone is not yet approved by most national medicines agencies outside of use for the treatment of T2DM. Hence, currently, off-label use of pioglitazone for NAFLD/NASH treatment requires the patient’s consent [85]. Concerns regarding weight gain (notably, a gain of 2–4% of body weight after 6–36 months of pioglitazone therapy was reported in most RCTs), fluid retention, and risk of bone fractures (mostly in women) or bladder cancer may restrict the use of pioglitazone in NAFLD patients. Lastly, pioglitazone exerts important cardiovascular benefits, decreasing the risk of myocardial infarction and stroke in patients with T2DM or prediabetes [94,95]. Given that compared to those without liver involvement, patients with NAFLD are at higher risk of developing cardiovascular disease, independent of the presence of T2DM, this cardiovascular-protective agent should be considered in NASH patients. Interestingly, accumulating evidence now indicates that specific genetic polymorphisms may explain some of the inter-individual variability in response to pioglitazone treatment in NASH patients. For instance, in a small pilot study of 55 participants from a randomized controlled trial designed to determine the efficacy of long-term pioglitazone treatment in patients with NASH (NCT00994682), Kawaguchi-Suzuki et al. reported that *ADORA1* (Adenosine A1 Receptor) rs903361 was associated with resolution of NASH and improvement in the ballooning score in patients receiving pioglitazone [96].

Agents able to promote a safe disposal of various metabolic substrates are under investigation, such as PPARα/δ (i.e., dual activity on both PPAR-α and PPAR-δ receptors) and PPARα/γ agonists [58,97]. Experimentally, the PPARα component enhances the oxidation of fatty acids, whereas the PPARδ component exhibits important anti-inflammatory effects [97]. A prototype of a PPARα/δ agonist is elafibrinor, which can improve insulin resistance and inflammation [97]. In a phase 2b study of 274 patients with biopsy-proven NASH who were randomly assigned to receive elafibranor (120 mg once daily) or placebo, elafibranor was superior in achieving the reversal of NASH without worsening of fibrosis (20% in elafibranor group vs. 11% in placebo group, *p* = 0.018) [65]. A post hoc analysis of this trial, based on a revised definition for the resolution of NASH, further corroborated these findings [65]. The efficacy and safety of this agent in patients with NASH and varying grades of fibrosis have also been evaluated in a phase 3 trial, namely RESOLVE-IT (NCT02704403) [58]. However, the interim analysis failed to show the achievement of the primary histological end point of NASH resolution without worsening of fibrosis, and, consequently, this trial has been stopped (68) (For additional details please see: https://ir.genfit.com/news-releases/news-release-details/genfit-announces-results-interim-analysis-resolve-it-phase-3/ (accessed date: 20 February 2021). 

Experimental animal models of NASH now indicate that the PPARα/γ dual agonist saroglitazar may have beneficial effects on the liver [58]. A meta-analysis of 18 studies demonstrated that in patients with diabetic dyslipidaemia, saroglitazar was able to decrease serum ALT concentrations and improve cardiometabolic profiles [98]. Encouraging results on the potential role of saroglitazar in NASH patients come from a preliminary analysis of a randomized, double-blind, phase 2 trial with non-invasive liver end points (EVIDENCES II; NCT03061721) [58]. Ongoing clinical trials indicate that dual- and pan-PPAR agonists might have beneficial effects on NASH by interrelated mechanisms [58].

### 1.7. GLP-1 Receptor Agonists

Glucagon-like peptide 1 receptor agonists (GLP-1 RAs) are a class of glucose-lowering drugs able to induce significant weight loss (on average 3–5 kg) and improve insulin resistance [84,85]. GLP-1 receptors have been documented in mice and human hepatocytes, and the activation of such receptors may promote the reduction of hepatic steatosis by improving insulin-signaling pathways, hepatocyte lipotoxicity, and mitochondrial function [56,99]. For these reasons, GLP-1RAs have also been investigated as a therapeutic option for NASH. In a 2021 meta-analysis of 11 placebo-controlled or active-controlled phase 2 RCTs (including a total of nearly 950 middle-aged individuals) that used liraglutide, exenatide, dulaglutide, or semaglutide to specifically treat NAFLD or NASH, as detected by liver biopsy or imaging techniques, Mantovani et al. documented that treatment with GLP-1 RAs for a median of 26 weeks was associated with significant reductions in the absolute percentage of liver fat content, as assessed on magnetic resonance imaging (pooled weighted mean difference (WMD): −3.92%, 95% confidence interval −6.27% to −1.56%; I^2^ = 97%) and serum liver enzyme levels (mostly ALT), as well as with greater histological resolution of NASH without worsening of liver fibrosis (pooled random-effects odds ratio 4.06, 95% confidence interval 2.52–6.55; I^2^ = 0%; for liraglutide and semaglutide only) [66]. In the context of various specific RCTs available so far, liraglutide (which is a long-acting GLP-1RA) was evaluated in patients with either biochemistry-based or imaging-defined NAFLD by the LEAD (Liraglutide Effect and Action in Diabetes) program and LEAD-2 study [67] and in patients with biopsy-proven NASH by the LEAN (Liraglutide Efficacy and Action in NASH) trial [68]. Evidence from these studies documented that liraglutide improved serum liver enzyme levels and other metabolic end points (e.g., peripheral, hepatic, and adipose tissue insulin resistance), as well as promoted the improvement of hepatic steatosis and the resolution of NASH and hepatocyte ballooning [67,68]. Conversely, liraglutide failed to improve liver fibrosis [68]. The benefits of liraglutide on the aforementioned histological liver outcomes may likely be due to weight loss and its direct hepatic effect, thereby suggesting a possible synergistic and multifactorial effect [56]. GLP-1RAs are generally well tolerated and, among the RCTs available so far, they have a similar adverse event profile to placebo (or reference therapy), with the exception of an increased frequency of gastrointestinal symptoms, including loss of appetite, nausea, constipation, or diarrhea [56]. These events are, however, transient and mild-to-moderate in severity [56]. Studies of semaglutide or dulaglutide, two GLP-1 RAs requiring only weekly dosing, have produced interesting results [69,70]. In particular, in a 72-week, double-blind phase 2 trial of 320 patients with biopsy-confirmed NASH and liver fibrosis randomly assigned to receive semaglutide at a dose of 0.1 mg (80 patients), 0.2 mg (78 patients), or 0.4 mg (82 patients), or to receive placebo (80 patients), Newsome et al. reported that the percentage of patients in whom NASH resolution was observed with no worsening of fibrosis was 40% in the 0.1 mg group, 36% in the 0.2 mg group, 59% in the 0.4 mg group, and 17% in the placebo group (*p* < 0.05) [71]. Conversely, this trial did not report a significant between-group difference in the percentage of patients with an improvement in fibrosis stage (*p* = 0.48) [71]. Notably, in this RCT, participants were randomly assigned to receive once-daily semaglutide at a dose of 0.1 mg, 0.2 mg, 0.4 mg/day, or placebo [71], At present, the approved dosage of semaglutide for the treatment of T2DM is different [66]. Hence, the transferability of these findings [71] to clinical practice is currently uncertain. Given that liraglutide was reported to reduce the risk of cardiovascular events in patients with T2DM in the LEADER trial [100] and in a 2019 meta-analysis [101], in addition to other GLP-1 RAs, it is reasonable to suppose that liraglutide (along with other GLP-1RAs [69,70]) might become an important treatment option in NAFLD patients, especially if they are obese or have T2DM. In addition, therapy with GLP-1 RAs in combination with a glucagon inhibitory peptide, which is able to improve intestinal barrier function, is under study (NCT03437720) [97].

### 1.8. SGLT-2 Inhibitors

Sodium-glucose cotransporter-2 (SGLT-2) inhibitors are a relatively new class of glucose-lowering agents that increase glucose reabsorption by the kidneys and also by the bowel and heart [84,85]. SGLT-2 is particularly expressed on the renal epithelial cells edging the S1 segment of the proximal convoluted tubule and promotes glycosuria [84,85]. In this regard, the regulation of blood glucose control is independent of insulin secretion [84,85]. Experimental studies using animal NASH models suggested a favorable effect of SGLT-2 inhibitors on liver steatosis, inflammation, and fibrosis owing to a combination of negative energy balance by increased glycosuria and substrate switching toward lipids as a source of energy expenditure [102]. For these reasons, SGLT-2 inhibitors have also been investigated as a therapeutic option for NASH in humans. In a 2021 meta-analysis of 12 RCTs evaluating the efficacy of dapagliflozin, empagliflozin, ipragliflozin, or canagliflozin to treat NAFLD for a median period of 24 weeks with a total of 850 middle-aged overweight or obese individuals with NAFLD, Mantovani et al. reported that, compared to placebo or reference therapy, treatment with SGLT-2 inhibitors decreased serum ALT (pooled WMD: −10.0 IU/L, 95% confidence interval −12.2 to −7.8 IU/L; I^2^ = 11%) and GGT levels (pooled WMD: –14.5 IU/L, 95% confidence interval −19.4 to −9.6 IU/L, I^2^ = 39%), as well as the absolute percentage of liver fat content on magnetic resonance imaging (pooled WMD: −2.1%, 95% confidence interval −2.6 to −1.5%; I^2^ = 0%) [72]. Similar findings were also reported in another meta-analysis [73]. However, it is important to highlight that most of the RCTs available so far are small and do not test the effect of SGLT-2 inhibitors on liver histology. With regard to adverse effects across the published RCTs, SGLT-2 inhibitors have a similar adverse event profile to placebo (or reference therapy), with the exception of a higher risk of genitourinary infections [56]. Given that SGLT-2 inhibitors have documented relevant cardio-renal benefits in large RCTs enrolling patients with and without T2DM [103,104], they are attractive agents for patients with NAFLD. At present, there are additional ongoing RCTs testing the effects of SGLT-2 inhibitors in these patients.

### 1.9. Statins and Other Lipid-Lowering Agents

Seeing that NAFLD is associated with specific features of MetS, including T2DM, hypertension, obesity, and (atherogenic) dyslipidemia, clinicians frequently have to manage these conditions in NAFLD patients. Abnormal blood cholesterol levels can be controlled by statins, known to inhibit 3-hydroxy-3-methylglutaryl-coenzyme A reductase (HMG-CoA), which is a key enzyme implicated in cholesterol synthesis [105]. Along with their lipid-lowering effect, statins also exhibit pleiotropic properties, including antioxidative and anti-inflammatory effects, neoangiogenesis, and improvement of endothelial functions [105]. Although statins might elevate aminotransferase levels, liver damage owing to this lipid-lowering agent is infrequently observed in clinical practice [105,106]. In this way, it is estimated that an elevation of liver enzymes >3 times the upper limit of normal can be observed in <1% of patients treated with statins [105,107]. For this reason, to date, the periodic monitoring of transaminase levels is no longer recommended [105]. In addition, NAFLD patients are at high risk of cardiovascular morbidity and mortality [5,6,30]. In a post hoc analysis of the Greek Atorvastatin and Coronary Heart Disease Evaluation (GREACE) study, enrolling 437 patients with moderately abnormal liver tests at baseline due to the presence of NAFLD (227 of whom were treated with a statin and 210 were not), Athyros et al. reported that NAFLD patients who received statins had significantly reduced cardiovascular morbidity, without significant liver-related adverse events [108]. Interestingly, accumulating evidence also suggests that in patients with NAFLD, statin treatment is associated with a significant improvement of liver steatosis, inflammation, and even fibrosis [109,110,111]. For instance, in a recent observational study of 11,593,409 individuals from the National Health Information Database of the Republic of Korea (712,262 of whom had a fatty liver index >60, which is indicative of NAFLD), Lee et al. reported that the use of statins was associated with a reduced risk of NAFLD (adjusted odds ratio 0.66, 95% confidence interval 0.65–0.67), as well as with a reduced risk of significant liver fibrosis, indirectly evaluated by BARD score, even after controlling for several metabolic risk factors (adjusted odds ratio 0.43, 95% confidence interval 0.42–0.44) [110]. Experimental studies using animal models have shed light regarding the potential mechanisms by which statins might promote the improvement of liver histology in NAFLD patients. In experimental NASH, for instance, statins affect the paracrine signaling (including the RhoA/Rho-kinase pathway) of hepatocytes on hepatic stellate cells, thereby blocking hepatic stellate cell activation and, consequently, fibrosis processes [105]. In a bile duct ligated mouse model, the antifibrotic effect of statins was due to the reduction of serum bile acid levels [105]. In other models, including angiotensin-II-induced liver fibrosis, statins decreased fibrosis by reducing inflammatory activity [105].

All etiologies of chronic liver disease have a common end stage characterized by portal hypertension and liver remodeling. Statins may even modulate the pathways (e.g., RhoA/Rho-kinase and nitric oxide (NO)) involved in the impaired intrahepatic resistance and vascular tone regulation, causing portal hypertension [105]. Based on these data, statins seem to be able to modulate the dynamic and the structural components of chronic liver diseases (including fibrosis), making themselves useful in the management of patients with and without T2DM who have cirrhosis and portal hypertension [105,112].

Information regarding the effects of other lipid-lowering agents on liver histology in patients with NAFLD is also available. Ezetimibe is a lipid-lowering agent acting by decreasing cholesterol absorption in the intestines. Specifically, it blocks the critical mediator of cholesterol absorption—namely, the Niemann–Pick C1-like 1 (NPC1L1) protein—on the gastrointestinal tract epithelial cells, as well as in hepatocytes. In a small meta-analysis of six studies (two RCTs and four single-arm trials) for a total 273 NAFLD patients with and without T2DM, it was reported that ezetimibe significantly reduced serum liver enzyme levels as well as improved hepatic steatosis and hepatocyte ballooning [113]. However, in that study, ezetimibe did not improve liver fibrosis [113].

Fenofibrate, which is a PPAR-α agonist, seems to not reduce liver fat content in NAFLD patients with and without T2DM [114].

Omega-3 polyunsaturated fatty acids (n-3 PUFAs) contain several long chain fatty acids, such as α-linolenic acid (α-ALA), stearidonic acid (SDA), eicosapentaenoic acid (EPA), docosapentaenoic acid (DPA), and docosahexaenoic acid (DHA) [115]. EPA and DHA decrease the levels of triglyceride and very-low-density lipoproteins, which are converted to low-density lipoprotein and intermediate-density lipoprotein [115]. Accumulating evidence also suggests that dietary *n*−3 PUFAs could improve insulin resistance by regulating mitochondrial function and mediating anti-inflammatory effects [115]. Several preclinical studies using animal NASH models have suggested that the supplementation of *n*−3 PUFAs could exert a positive impact on NAFLD by diminishing hepatic fat deposition and preventing the proinflammatory state [116]. For these reasons, *n*−3 PUFAs have been investigated as a therapeutic option for NASH in humans. In a 2020 meta-analysis of 22 RCTs with a total of 1366 participants with and without T2DM, Lee et al. reported that *n*−3 PUFAs supplementation significantly reduced liver fat (evaluated by imaging methods) when compared with placebo (pooled risk ratio 1.52, 95% confidence interval 1.09–2.13) [115]. In that study, in addition, *n*−3 PUFAs supplementation significantly improved the levels of triglyceride, total cholesterol, high-density lipoprotein, and BMI [115]. Similar findings were also observed in another meta-analysis [117]. However, it is important to point out that the size of the effect of *n*−3 PUFAs is relatively small and that the optimal dose and duration of treatment with them are not yet established [117]. Hence, we believe that additional well-designed randomized clinical trials are needed to recommend omega-3 PUFA supplementation for the treatment of NAFLD in patients with and without T2DM. In this regard, based on the current evidence, NICE guidelines [15] do not recommend the supplementation with *n*−3 PUFAs in NAFLD patients (Table 1).

Proprotein convertase subtilisin kexin type-9 (PCSK-9) is a key regulator of cholesterol homeostasis, acting as a potent inhibitor of the LDL receptor (LDLR) pathway [118]. Once secreted by the hepatocyte, circulating PCSK-9 binds to the extra-cellular EGF(A) domain of the LDLR and promotes its lysosomal degradation [118]. Accumulating data suggests that high intrahepatic or circulating PCSK-9 levels increase muscle and liver lipid storage, adipose energy storage, hepatic fatty acids, and triglycerides storage, thereby promoting the development of NAFLD [118,119]. Preliminary evidence indicates that antisense particles against PCSK-9 mRNA or anti-PCSK-9 antibodies, able to reduce circulating PCSK-9 levels, might improve NAFLD independent of LDL cholesterol level reduction [119].

### 1.10. Anti-Hypertensive Agents: Spotlight on ACEi and ARBs

Experimental and clinical studies, although not all, have demonstrated that angiotensin converting enzyme inhibitors (ACEi) or angiotensin II receptor blockers (ARBs) may have an anti-fibrotic effect in the liver. In animal NASH models, for instance, the treatment with ACEi and ARBs can switch off the profibrogenic state and lead to regression of fibrosis [120,121,122,123]. Clinical studies have been conducted to test the potential anti-fibrotic effects of these drugs in NAFLD patients, with inconsistent results [124,125,126,127]. In addition, trials enrolling exclusively T2DM patients are absent to date. In a randomized, open-label trial of 137 individuals with biopsy-proven NASH (approximately 20% with established T2DM at baseline) and randomly assigned to receive either 4 mg twice daily of rosiglitazone, 4 mg of rosiglitazone and 500 mg of metformin twice daily, or 4 mg of rosiglitazone twice daily and 50 mg of losartan once daily for 48 weeks, combination therapy with rosiglitazone and metformin or rosiglitazone and losartan conferred no higher benefit than rosiglitazone alone on histopathological features [126]. A double-blind randomized-controlled trial of losartan (50 mg once a day) vs. placebo for 96 weeks in 45 patients with histological evidence of NASH (approximately 60% with established T2DM at baseline) failed to recruit sufficient patients to determine whether losartan had anti-fibrotic effects in the liver [127]. Therefore, to date, given the current evidence, it is possible to speculate that some ARBs (especially losartan) may be beneficial in treating NASH/NAFLD and its consequences, but additional and larger controlled clinical trials are required to provide consistent data on this topic, especially in those with T2DM.

### 1.11. Anti-Platelet Aggregation Agents

There are few data from prospective studies on the effects of aspirin on fibrosis in patients with NAFLD. In a recent observational study of patients with biopsy-proven NAFLD, daily aspirin use was associated with less severe histologic features of NAFLD and NASH, as well as with lower risk for progression to advanced fibrosis with time [74]. This evidence was supported by some studies showing that platelet-derived GPIbα may play a role in the development of NASH, independent of von Willebrand factor (vWF), *p*-selectin, and Mac-1 (also known as integrin α_M_β_2_, or CD11b/CD18) [75]. These mechanisms might offer a novel potential target against NASH [75].

### 1.12. Vitamin E

Vitamin E is a potent antioxidant agent and may be useful in the treatment of NAFLD. Experimentally, vitamin E exerts beneficial effects on NAFLD in animal NASH models by multiple mechanisms, including the improvement of lipid and glucose metabolism with the activation of the Nrf2/CES1 signaling pathway [128] and the reduction of oxidative stress via the downregulation of iNOS and nicotinamide adenine dinucleotide phosphate (NADPH) oxidase [129]. The PIVENS trial, enrolling 247 adults with NASH randomly assigned to receive vitamin E at a dose of 800 U/day or placebo over 96 weeks, documented significant improvements in serum liver enzymes and in some histological features of NASH (i.e., steatosis, inflammation, and ballooning) in the vitamin E group [59]. That said, at present, insufficient data are available to use vitamin E in NAFLD patients with T2DM. The AASLD and NICE practice guidelines [2,15] for the management of NAFLD support the use of vitamin E in non-diabetic patients with NASH, especially in secondary/tertiary settings. However, we believe that additional evidence is required to support the use of this agent, especially long term.

## 2. New Drugs for NAFLD/NASH

In recent years, several other potential agents have been tested for NAFLD [97]. However, it is important to note that in most of the trials available so far, only a subset of NASH patients had established T2DM at baseline.

Synthetic ligands that activate the nuclear receptor, the farnesoid X receptor (FXR), improve insulin resistance, regulate glucose and lipid metabolism, and have direct anti-inflammatory and anti-fibrotic effects in animal NASH models [97,130]. Obeticholic acid (OCA), which is a synthetically modified analogue of chenodeoxycholic acid, is the prototype for this class of agents [97]. The multicenter, randomized, placebo-controlled FLINT trial, enrolling 283 individuals with non-cirrhotic NASH (approximately 50% with established T2DM), documented that OCA improved histological features of NASH, including fibrosis [76]. In the 18 month interim analysis of the multicenter, randomized, placebo-controlled phase 3 trial REGENERATE study that evaluated the safety and efficacy of two doses of OCA (10 mg or 25 mg daily) relative to placebo in 931 patients (mean age 55 years; 58% females; 57% with established T2DM) with biopsy-proven stage F2–F3 fibrosis, Yonoussi et al. reported that improvement of fibrosis was obtained in 71 (23%) of 308 patients in the OCA 25 mg group, compared with 37 (12%) of 311 patients in the placebo group (*p* = 0.0002) [77]. Obeticholic acid has side-effects, such as pruritus and elevated LDL cholesterol levels [77]. In particular, the effect of OCA on the lipid profile is clinically relevant, as NAFLD patients have an increased risk of CVD, which is, of note, the principal cause of death in these patients. In the REGENERATE trial, LDL cholesterol levels increased by nearly 20% from baseline, although it was suggested that the increase in LDL cholesterol levels tended to be transient and controlled by statin treatment [77]. In another study enrolling 196 patients (99 in OCA group and 97 in placebo group) from the FLINT trial, OCA therapy was associated with increases in small VLDL particles, large and small LDL particles, and a reduction in HDL particles at 12 weeks [131]. Such alterations in lipoprotein concentrations reverted to baseline after drug discontinuation [131]. The safety of the combination of OCA with atorvastatin in NASH patients is being tested in the randomized, placebo-controlled, double-blind “combination OCA and statins for monitoring of lipids (CONTROL)” phase 2 study (NCT02633956) [132]. Analogously to pioglitazone, a pilot genome-wide association study using FLINT participants showed that some genetic variants may be associated with histological improvement in NASH patients receiving OCA [133]. Among the tested genetic variants, rs75508464 variant near CELA3B (chymotrypsin-like elastase 3B) gene seems to document a significant effect on NASH resolution in patients receiving OCA [133].

Non-bile acid farnesoid X activated receptor (FXR) agonists, such as tropifexor, cilofexor, EDP-305, and nidufexor, are under evaluation for the potential to not increase LDL cholesterol levels (or other lipoproteins) and to not cause pruritus [97].

Another important pathway able to enhance FXR activity is the release of growth factor FGF-19 from the intestine upon bile acid binding to FXR, documenting favorable effects in in animal NASH models [97]. In phase 2a study (NCT02443116), the FGF-19 analog—namely, NGM282—achieved an important reduction in hepatic fat content and liver enzymes in 166 patients with biopsy-proven NASH (mean age 52 years; approximately 60% with established T2DM) [134]. A recent study confirmed and extended these findings [135].

Thyroid hormone receptor (THR)-β-selective agonists has been tested to reduce lipotoxic load in the liver in animal NASH models [97]. Importantly, its utilization improves circulating lipids in healthy humans [97]. In a multicenter, randomized, double-blind, placebo-controlled, phase 2 trial of 125 NAFLD patients (mean age 50 years; 50% men; 39% with established T2DM), resmetirom (also called MGL-3196)—a liver-directed, orally active, selective thyroid hormone receptor-β agonist—showed a relative reduction of hepatic fat (evaluated by MRI proton density fat fraction), when compared with placebo, at week 12 (−32.9% resmetirom vs. −10.4% placebo; *p* < 0.0001) and at week 36 (−37.3% resmetirom vs. −8.5% placebo; *p* < 0.0001) [79]. Transient mild diarrhea and nausea were more frequently observed in the resmetirom group than the placebo group [79].

Diacylglycerol-O-acyltransferase 2 (DGAT2) is one of two enzyme isoforms (i.e., DGAT1 and DGAT2) that catalyze the final step of triglyceride synthesis by promoting the linkage of diacylglycerol to acyl-coenzyme A [78,136]. Whilst DGAT1 is highly expressed in the small intestine, DGAT2 is mostly expressed in the liver [78]. Preliminary evidence from animal NASH models has reported that antisense inhibition of DGAT2 can decrease triglyceride synthesis and hepatic triglyceride levels, as well as improve hepatic steatosis and plasma lipoprotein profiles [78,136]. IONIS-DGAT2Rx is a 2′-*O*-methoxyethyl chimeric antisense oligonucleotide inhibitor that mediates enzyme-mediated degradation of DGAT2 mRNA in order to prevent production of DGAT2 protein [78,136]. In a recent small randomized, placebo-controlled phase 2 trial of IONIS-DGAT2Rx enrolling 44 NAFLD patients with T2DM, randomly assigned to receive IONIS-DGAT2Rx (29 patients) or placebo (15 patients) for a total of 13 weeks, Loomba et al. showed that the mean absolute reduction of liver fat content (as quantified by MRI-estimated proton density fat fraction) from baseline was −5.2% in the IONIS-DGAT2Rx group as compared with −0.6% in the placebo group (*p* = 0.026) [78]. However, six serious adverse events (e.g., acute exacerbation of chronic obstructive pulmonary disease, cardiac arrest, ischemic cerebral infarction, increased blood triglycerides, deep-vein thrombosis, acute pancreatitis) occurred in four patients in the IONIS-DGAT2Rx group, whereas no serious adverse events were reported in the placebo group [78].

The innate and adaptive immune systems are implicated in the pathogenesis of NASH [97]. In this regard, based on evidence from animal models, the C–C motif chemokine receptor 2 (CCR2)-CCR5 chemokine axis increases the innate immune response into the liver, as well as promotes the activation of hepatic stellate cells, which are the key producers of collagen, thereby leading to fibrosis [97,137]. In a small clinical trial of 289 NASH patients (mean age 54 years; 52% women; 50% with established T2DM), the inhibition of CCR2-CCR5 with cenicriviroc reduced short-term fibrosis progression [80]. A phase 3 trial is currently ongoing (NCT03028740) [97].

Some experimental studies have focused on the specific inhibition of the fibrosis process into the liver [97,138]. A potential mechanism is the use of an inhibitory antibody to lysyl oxidase-2 (LOXL-2), which is an enzyme that chemically crosslinks fibrillary collagen [97,138]. In this context, LOXL-2 inhibition can enhance the macrophage-mediated collagen degradation in animal NASH models [97,138]. However, in two phase 2b trials of 219 patients with bridging fibrosis caused by NASH (median age 57 years; approximately 68% with established T2DM) who were randomly assigned to groups given weekly subcutaneous injections of simtuzumab (a monoclonal antibody against LOXL-2) or placebo for 240 weeks, Harrison et al. reported that simtuzumab was ineffective in decreasing hepatic collagen content and hepatic venous pressure gradient [82].

Caspases are intracellular proteases that play a role in apoptotic cell death by cleavage of cytoskeletal proteins [97,139]. Apoptosis is increased in NASH patients and serum levels of cleaved keratin-18 is closely associated with liver fibrosis [97,139]. Emricasan is a pan-caspase inhibitor that had previously been shown to decrease caspase-3/7 activity and cleave keratin-18 and serum ALT levels in NAFLD patients [97]. However, a double-blind, placebo-controlled study enrolling 318 biopsy-proven NASH patients (approximately 50% with established T2DM) who were randomly assigned to emricasan or placebo for 72 weeks, reported that emricasan treatment did not improve liver histology and, importantly, may even have worsened fibrosis and ballooning in a subset of patients [81].

Apoptosis signal-regulating kinase 1 (ASK1) plays a key role in hepatocyte injury, inflammation, and fibrosis [97]. Experimental evidence from animal NASH models has suggested that the selective inhibition of ASK1 by selonsertib may have an important antifibrotic effect in NASH [97]. However, recently, two randomized, double-blind, placebo-controlled phase 3 trials of selonsertib in patients with NASH and bridging fibrosis (F3; STELLAR-3) or compensated cirrhosis (F4; STELLAR-4) failed to demonstrate a reduction of liver fibrosis with selonsertib treatment in these patients [83]. Are these two trials a defeat? No, but, according to Rinella and Noureddin [140], we believe that it is fundamental to stratify NAFLD patients carefully when we design the next trials.

A Bayesian network meta-analysis combining direct and indirect treatment comparisons has recently evaluated the comparative effectiveness of various pharmacological agents for the treatment of NASH [141]. Nine randomized, controlled trials with a total of nearly 1000 patients with biopsy-proven NASH comparing vitamin E, glitazones, pentoxifylline, or OCA to placebo or one another were found [141]. This study showed that pentoxifylline and OCA improved fibrosis, whereas vitamin E, glitazones, and OCA improved ballooning degeneration in NASH patients [141]. However, these data do not provide straightforward recommendations for drug treatment of NAFLD.

The intestinal microbiome has a key role in the development and progression of NAFLD [142]. At present, probiotics, prebiotics, and bovine colostrum containing antibodies to endotoxin are under evaluation for NASH [142,143]. For instance, in a double-blind phase 2 trial of 104 UK patients with NAFLD (mean age 51 years; 65% men; 37% with established T2DM) who were randomly assigned to receive synbiotic agents (i.e., fructo-oligosaccharides, 4 g twice per day, plus *Bifidobacterium animalis* subspecies *lactis* BB-12) or placebo for 14 months, Scorletti et al. reported that the administration of a synbiotic combination (probiotic plus prebiotic) altered the fecal microbiome but did not improve liver fat content (as measured by MRI) or indirect markers of liver fibrosis [144].

**Figure 2:**
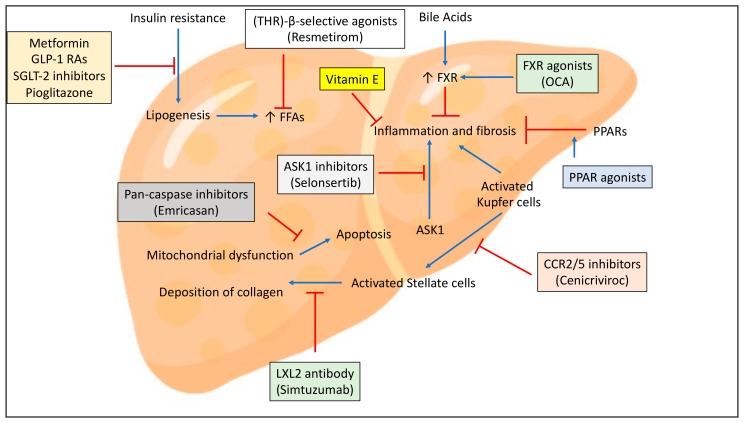
Main medications against NAFLD and its advanced forms and their mechanisms of action. See text for details. Note: Blue lines correspond to signal activation; red lines correspond to signal block. Abbreviations: ASK-1, apoptosis signal-regulating kinase-1; FFA, free fatty acids; FXR, farnesoid X receptor; GLP-1 RAs, glucagon-like peptide-1 receptor agonists; LOXL-2, lysyl oxidase-like 2; OCA, obeticholic acid; PPAR, peroxisome proliferator activated receptor; SGLT-2, sodium-glucose co-transporter-2; THR, thyroid hormone receptor.

Main agents against NAFLD and their mechanisms of action are summarized in Figure 2.

## 3. Conclusions

In spite of many advances in our knowledge regarding the epidemiology and pathogenesis of NAFLD, at present the only available and effective treatment for NAFLD and its advanced forms is weight loss. In addition, most RCTs available so far (with the exception of a phase 2 trial testing IONIS-DGAT2Rx [78]) have included patients with and without T2DM, and, consequently, the current information on new drugs (such as OCA, selonsertib, elafibranor, cenicriviroc, or resmetirom) as monotherapy for NAFLD treatment may not necessarily be generalizable to all patients with T2DM. In these patients, however, some glucose-lowering agents, such as pioglitazone, GLP-1RAs, and SGLT-2 inhibitors, may be useful for treating NAFLD.

A current challenge in the field of NAFLD is the lack of reliable and non-invasive endpoints for NASH [145] for use in the trials, although novel blood-based diagnostic tests to non-invasively rule in or rule out NASH risk are under way [114,146]. In addition, although the resolution of NASH and/or the improvement of fibrosis are currently accepted endpoints, histologic assessment using liver biopsy is often suboptimal and always invasive [145]. Importantly, to date, trials of drugs as monotherapy for the treatment of NASH have reported response rates from 30% to 50% as compared with placebo or reference therapy [145]. Considering the multiple pathways implicated in NASH pathogenesis, as well as the single response from single-agent therapies, it is reasonable to assume that a combination of different therapies might be more appropriate to treat NASH [145]. In this context, theoretically, the combination of two (or more) therapies might enhance the response rates, as well as might convert non-responders or partial responders to monotherapy into true responders. To date, there are ongoing trials regarding the treatment of NASH using multiple agents [145]. For instance, given that GLP-1 RAs are able to promote weight loss, the GLP-1 RA semaglutide is being investigated in combination with the FXR agonist cilofexor, as well as in combination with the Acetyl-CoA carboxylase inhibitor firsocostat, in a phase 2 proof-of-concept trial (NCT03987074) [145]. The FXR agonist tropifexor is also being investigated in combination with the SGLT-1/2 inhibitor licogliflozin in NASH patients with varying stages of liver fibrosis (NCT04065841) [145]. The phase 2 TANDEM trial is being testing the combination of cenicriviroc with 2 doses of tropifexor over 48 weeks in NASH patients (NCT03517540) [145].

Given the heterogeneity of NASH patients, it is also fundamental to identify appropriate individuals for a specific combination [17,145]. The identification of specific genetic polymorphisms might provide useful and important information regarding the response to treatment [145]. A single nucleotide polymorphism rs903361 in the *ADORA1* gene has been recently associated with the resolution of NASH in patients treated with pioglitazone [96,145]. The rs75508464 variant near *CELA3B* gene seems to be associated with the most important effect on the resolution of NASH in patients receiving OCA [133,145]. Additional studies are, however, required to improve our understanding of identifying which NAFLD patients would have a higher probability of treatment response with a specific agent as monotherapy or with a combination therapy.

## Figures and Tables

**Table 1:** Management of NAFLD according to EASL-EASD-EASO, AASLD and NICE guidelines.

	Diets	Physical Activity	Drugs
**EASL-EASD-EASO [1] Guidelines**	Diets that have a 500–1000 kcal/day deficit (i.e., diets containing 500–1000 kcal less than the person needs to stay the same weight) are recommended for weight loss (7–10% total weight loss target); limit alcohol consumption; no coffee drink limitation; prefer Mediterranean diet	Moderate intensity aerobic or exercise training (150–200 min/week) in 3–5 sessions	Prefer pioglitazone; notably, off-label use in absence of T2DM in most countries
**AASLD [2] Guidelines**	Prefer hypocaloric diet with a daily reduction by 500–1000 kcal; no specific diet to prefer; limit alcohol consumption	Moderate-intensity exercise (≥150 min/week)	Prefer pioglitazone in patients with T2DM; prefer vitamin E in those without T2DM
**NICE [15] Guidelines**	Diets that have a 600 kcal/day deficit (i.e., diets containing 600 kcal less than the person needs to stay the same weight) or that reduce calories by lowering the fat content (low-fat diets) are recommended for sustainable weight loss; limit alcohol consumption; no recommendation for supplementation with omega-3 fatty acids	At least 45 to 60 min of moderate-intensity activity a day (refer also to specific NICE guidelines for obesity)	Prefer pioglitazone in T2DM patients; prefer vitamin E in non-diabetic patients with advanced fibrosis

EASL-EASD-EASO: European Association for the Study of the Liver (EASL)—European Association for the Study of Diabetes (EASD)—European Association for the Study of Obesity (EASO); AASLD: American Association for the Study of Liver Diseases; NICE: National Institute for Health and Care Excellence; T2DM: type 2 diabetes mellitus.

**Table 2:** Main agents and their characteristics for treating NAFLD and its advanced forms.

Agents, Ref.	Mechanisms of Action	Relevant Studies	Primary Endpoint(s)	Main Side Effects	Comments
**Targets Related to Insulin Resistance and/or Lipid Metabolism**					
Metformin [56,57]	- AMP-activated protein kinase (AMPK)-dependent - Alterations in cellular energy charge - Modulation of the cellular redox state - Gluconeogenesis	- Multiple studies - TONIC trial	- Small beneficial effects on liver steatosis and inflammation - No effects on liver fibrosis and resolution of NASH	Lactic acidosis, diarrhea, nausea, vomiting, flatulence, vitamin B12 deficiency (long-term use)	- Not recommended for NAFLD treatment by most guidelines - Potential effect in reducing risk of cirrhosis and HCC
Pioglitazone [58,59,60,61,62,63,64]	- PPAR-gamma agonist	- PIVENS trial - Multiple studies - Meta-analyses	- Improvement in NAS >2 without fibrosis worsening - Improvement of fibrosis	Weight gain (usually 2–4% of body weight), fluid retention, bone fractures (mostly in women), bladder cancer	- Guidelines recommend this agent in patients with biopsy-proven NASH, regardless of the presence of T2DM - This agent is not yet approved by most national medicines agencies outside the treatment of T2DM - Off-label use for NASH treatment requires the patient’s consent - Cardiovascular benefits in patients with T2DM or prediabetes
Elafibrinor [58,65]	- PPARα/δ agonist	- RESOLVE-IT trial	- Failed to achieve NASH resolution, without a worsening of fibrosis	Nausea, headache, diarrhea, fatigue, asthenia, renal failure, abdominal pain, vomiting, myalgia, rash, pruritus	- RESOLVE-IT has been stopped
GLP-1 RAs (mainly liraglutide and semaglutide) [56,66,67,68,69,70,71]	- GLP-1 receptor agonist	- LEAN trial - LEAD study - LEAD-2 study - Phase 2 trials - Meta-analyses	- Resolution of NASH - Improvement of hepatic steatosis, hepatocyte ballooning - No effects on liver fibrosis	Loss of appetite, nausea, constipation, diarrhea	- Premature to consider in NASH patients - Relevant cardio-renal benefits in large RCTs enrolling patients with T2DM
SGLT-2 inhibitors (dapagliflozin, empagliflozin, canagliflozin, ipragliflozin) [56,72,73]	- Inhibition of SGLT-2 that is highly expressed on the renal epithelial cells edging the S1 segment of the proximal convoluted tubule	- Multiple studies - Meta-analyses	- Improvement of serum liver enzymes (especially ALT) - Improvement of liver fat content	Genitourinary infections, diabetic ketoacidosis, hypotension	- Premature to consider in NASH patients - No current trials with histological end-points are available - Relevant cardio-renal benefits in large RCTs enrolling patients with and without T2DM
Acetylsalicylic acid (ASA) [74,75]	- Platelet cargo, platelet adhesion, platelet activation	- Experimental studies	- Improvement of fibrosis	Hemorragia	- No RCTs
Obeticholic acid (OCA) [76,77]	- FXR (farnesoid X receptor) ligand	- REGENERATE trial - FLINT trial	- Resolution of NASH without worsening fibrosis - Improvement of fibrosis without worsening NASH	Pruritus, increased LDL cholesterol levels and other lipoproteins	- Might be considered in selected NASH patients - Concerns about increased LDL cholesterol levels - The safety of the combination of OCA with atorvastatin in NASH patients is being tested in the CONTROL study (NCT02633956)
IONIS-DGAT2_Rx_ [78]	- 2′-O-methoxyethyl chimeric antisense oligo-nucleotide inhibitor that mediate enzyme-mediated degradation of DGAT2 mRNA	- Phase 2 trial	- Reduction of liver fat content (as quantified by MRI-estimated proton density fat fraction)	Serious adverse events occurred in 4 patients in the IONIS-DGAT2_Rx_ group	- Preliminary data - The phase 2 trial included NAFLD patients with T2DM - It is being testing in combination therapy trials (e.g., NCT03776175)
Resmetirom [79]	- Thyroid hormone receptor (THR)-β-selective agonists	MAESTRO-NASH trial	- Reduction of liver fat content (as quantified by MRI-estimated proton density fat fraction)	Mild diarrhea, nausea	- Preliminary data
**Targets related to lipotoxicity and oxidative stress**					
Vitamin E [59]	- Antioxidant	- PIVENS trial	- Improvement in NAS > 2 without fibrosis worsening	Nausea, diarrhea, intestinal cramps, fatigue, weakness, headache, blurred vision, rash, gonadal dysfunction, increased concentration of creatine in the urine (creatinuria), prostate cancer	- May be used in non-diabetic adults with biopsy-proven NASH
**Targets related to inflammation and immune activation**					
Cenicriviroc [80]	- CCR2/5 inhibitor	- AURORA trial - CENTAUR trial	- Improvement in fibrosis without worsening NASH	Headache, nausea, constipation, diarrhea, sinusitis	- Premature to consider in NASH patients - It is being testing in combination therapy trials (e.g., NCT03517540)
**Targets related to cell death (e.g., apoptosis and necrosis)**					
Emricasan [81]	- Pan-caspase inhibitor	- ENCORE-NF trial - ENCORE-PH trial - ENCORE-LF trial	- Failed to improve liver fibrosis	Headache, nausea, fatigue	- None
**Targets related to fibrogenesis or collagen turnover**					
Simtuzumab [82]	- Monoclonal antibody against LOXL-2 (lysyl oxidase-like 2)	- Two phase 2b trials	- Ineffective in decreasing hepatic collagen content and hepatic venous pressure gradient	Headache, increased lipase levels	- None
Selonsertib [83]	- ASK-1 (Apoptosis signal-regulating kinase 1) inhibitor	- STELLAR 3 - STELLAR 4	- Failed to improve liver fibrosis	Constipation, acute kidney injury, back pain, hyperglycemia (∼10% of cases), cellulitis, gout, hypertriglyceridemia (∼10% of cases)	- Drug will continue to be used in phase 2 combination therapy trials (e.g., NCT02781584; NCT03449446)

Abbreviations: ALT, alanine aminotransferase; GLP-1, glucagon-like peptide-1; MRI, magnetic resonance imaging; NAS, NAFLD Activity Score; NASH, non-alcoholic steatohepatitis; PPAR, peroxisome proliferator-activated receptor; SGLT-2, sodium-glucose co-transporter-2; T2DM, type 2 diabetes.

## References

[1] European Association for the Study of the Liver (EASL), European Association for the Study of Diabetes (EASD), European Association for the Study of Obesity (EASO) (2016). EASL–EASD–EASO Clinical Practice Guidelines for the management of non-alcoholic fatty liver disease. J. Hepatol..

[2] Chalasani N., Younossi Z., LaVine J.E., Charlton M., Cusi K., Rinella M., Harrison S.A., Brunt E.M., Sanyal A.J. (2018). The diagnosis and management of nonalcoholic fatty liver disease: Practice guidance from the American Association for the Study of Liver Diseases. Hepatology.

[3] Younossi Z.M., Koenig A.B., Abdelatif D., Fazel Y., Henry L., Wymer M. (2016). Global epidemiology of nonalcoholic fatty liver disease-Meta-analytic assessment of prevalence, incidence, and outcomes. Hepatology.

[4] Younossi Z.M., Golabi P., de Avila L., Paik J.M., Srishord M., Fukui N., Qiu Y., Burns L., Afendy A., Nader F. (2019). The global epidemiology of NAFLD and NASH in patients with type 2 diabetes: A systematic review and meta-analysis. J. Hepatol..

[5] Mantovani A., Scorletti E., Mosca A., Alisi A., Byrne C.D., Targher G. (2020). Complications, morbidity and mortality of nonalcoholic fatty liver disease. Metabolism.

[6] Lonardo A., Nascimbeni F., Mantovani A., Targher G. (2018). Hypertension, diabetes, atherosclerosis and NASH: Cause or consequence?. J. Hepatol..

[7] Targher G., Lonardo A., Byrne C.D. (2018). Nonalcoholic fatty liver disease and chronic vascular complications of diabetes mellitus. Nat. Rev. Endocrinol..

[8] Anstee Q.M., Targher G., Day C.P. (2013). Progression of NAFLD to diabetes mellitus, cardiovascular disease or cirrhosis. Nat. Rev. Gastroenterol. Hepatol..

[9] Mantovani A., Byrne C.D., Bonora E., Targher G. (2018). Nonalcoholic Fatty Liver Disease and Risk of Incident Type 2 Diabetes: A Meta-analysis. Diabetes Care.

[10] Ballestri S., Zona S., Targher G., Romagnoli D., Baldelli E., Nascimbeni F., Roverato A., Guaraldi G., Lonardo A. (2016). Nonalcoholic fatty liver disease is associated with an almost twofold increased risk of incident type 2 diabetes and metabolic syndrome. Evidence from a systematic review and meta-analysis. J. Gastroenterol. Hepatol..

[11] Eslam M., Newsome P.N., Sarin S.K., Anstee Q.M., Targher G., Romero-Gomez M., Zelber-Sagi S., Wong V.W.-S., Dufour J.-F., Schattenberg J.M. (2020). A new definition for metabolic dysfunction-associated fatty liver disease: An international expert consensus statement. J. Hepatol..

[12] Eslam M., Sanyal A.J., George J., International Consensus Panel (2020). MAFLD: A Consensus-Driven Proposed Nomenclature for Metabolic Associated Fatty Liver Disease. Gastroenterology.

[13] Polyzos S.A., Kang E.S., Tsochatzis E.A., Kechagias S., Ekstedt M., Xanthakos S., Lonardo A., Mantovani A., Tilg H., Côté I. (2020). Commentary: Nonalcoholic or metabolic dysfunction-associated fatty liver disease? The epidemic of the 21st century in search of the most appropriate name. Metabolism.

[14] Mantovani A., Dalbeni A. (2020). NAFLD, MAFLD and DAFLD. Dig. Liver Dis..

[15] Glen J., Floros L., Day C., Pryke R. (2016). Non-alcoholic fatty liver disease (NAFLD): Summary of NICE guidance. BMJ.

[16] Petroni M.L., Brodosi L., Bugianesi E., Marchesini G. (2021). Management of non-alcoholic fatty liver disease. BMJ.

[17] Mantovani A. (2019). Not all NAFLD patients are the same: We need to find a personalized therapeutic approach. Dig. Liver Dis..

[18] Romero-Gómez M., Zelber-Sagi S., Trenell M. (2017). Treatment of NAFLD with diet, physical activity and exercise. J. Hepatol..

[19] Wong V.W.-S., Wong G.L.-H., Chan R.S.-M., Shu S.S.-T., Cheung B.H.-K., Li L.S., Chim A.M.-L., Chan C.K.-M., Leung J.K.-Y., Chu W.C.-W. (2018). Beneficial effects of lifestyle intervention in non-obese patients with non-alcoholic fatty liver disease. J. Hepatol..

[20] Charatcharoenwitthaya P., Karaketklang K., Aekplakorn W. (2020). Cigarette Smoking Increased Risk of Overall Mortality in Patients with Non-alcoholic Fatty Liver Disease: A Nationwide Population-Based Cohort Study. Front. Med..

[21] Vos M.B., LaVine J.E. (2013). Dietary fructose in nonalcoholic fatty liver disease. Hepatology.

[22] Zelber-Sagi S., Salomone F., Mlynarsky L. (2017). The Mediterranean dietary pattern as the diet of choice for non-alcoholic fatty liver disease: Evidence and plausible mechanisms. Liver Int..

[23] Meir A.Y., Rinott E., Tsaban G., Zelicha H., Kaplan A., Rosen P., Shelef I., Youngster I., Shalev A., Blüher M. (2021). Effect of green-Mediterranean diet on intrahepatic fat: The DIRECT PLUS randomised controlled trial. Gut.

[24] Parra-Vargas M., Rodriguez-Echevarria R., Jimenez-Chillaron J.C. (2020). Nutritional Approaches for the Management of Nonalcoholic Fatty Liver Disease: An Evidence-Based Review. Nutrients.

[25] Zadeh S.H., Mansoori A., Hosseinzadeh M. (2020). Relationship between dietary patterns and non-alcoholic fatty liver disease: A systematic review and meta-analysis. J. Gastroenterol. Hepatol..

[26] Willett W.C., Sacks F., Trichopoulou A., Drescher G., Ferro-Luzzi A., Helsing E., Trichopoulos D. (1995). Mediterranean diet pyramid: A cultural model for healthy eating. Am. J. Clin. Nutr..

[27] Van Horn L., Carson J.A.S., Appel L.J., Burke L.E., Economos C., Karmally W., Lancaster K., Lichtenstein A.H., Johnson R.K., Thomas R.J. (2016). Recommended Dietary Pattern to Achieve Adherence to the American Heart Association/American College of Cardiology (AHA/ACC) Guidelines: A Scientific Statement from the American Heart Association. Circulation.

[28] Estruch R., Ros E., Salas-Salvadó J., Covas M.-I., Corella D., Arós F., Gómez-Gracia E., Ruiz-Gutiérrez V., Fiol M., Lapetra J. (2018). Primary Prevention of Cardiovascular Disease with a Mediterranean Diet Supplemented with Extra-Virgin Olive Oil or Nuts. N. Engl. J. Med..

[29] American Diabetes Association (2018). 5. Lifestyle Management: Standards of Medical Care in Diabetes—2019. Diabetes Care.

[30] Targher G., Day C.P., Bonora E. (2010). Risk of Cardiovascular Disease in Patients with Nonalcoholic Fatty Liver Disease. N. Engl. J. Med..

[31] Ryan M.C., Itsiopoulos C., Thodis T., Ward G., Trost N., Hofferberth S., O’Dea K., Desmond P.V., Johnson N.A., Wilson A.M. (2013). The Mediterranean diet improves hepatic steatosis and insulin sensitivity in individuals with non-alcoholic fatty liver disease. J. Hepatol..

[32] Bozzetto L., Prinster A., Annuzzi G., Costagliola L., Mangione A., Vitelli A., Mazzarella R., Longobardo M., Mancini M., Vigorito C. (2012). Liver Fat Is Reduced by an Isoenergetic MUFA Diet in a Controlled Randomized Study in Type 2 Diabetic Patients. Diabetes Care.

[33] Properzi C., O’Sullivan T.A., Sherriff J.L., Ching H.L., Jeffrey G.P., Buckley R.F., Tibballs J., MacQuillan G.C., Garas G., Adams L.A. (2018). Ad Libitum Mediterranean and Low-Fat Diets Both Significantly Reduce Hepatic Steatosis: A Randomized Controlled Trial. Hepatology.

[34] Targher G., Byrne C.D. (2018). Ad Libitum Mediterranean or Low-Fat Diets as Treatments for Nonalcoholic Fatty Liver Disease?. Hepatol..

[35] Wang D.D., Nguyen L.H., Li Y., Yan Y., Ma W., Rinott E., Ivey K.L., Shai I., Willett W.C., Hu F.B. (2021). The gut microbiome modulates the protective association between a Mediterranean diet and cardiometabolic disease risk. Nat. Med..

[36] Marchesini G., Petta S., Grave R.D. (2015). Diet, weight loss, and liver health in nonalcoholic fatty liver disease: Pathophysiology, evidence, and practice. Hepatology.

[37] Bacchi E., Negri C., Targher G., Faccioli N., Lanza M., Zoppini G., Zanolin E., Schena F., Bonora E., Moghetti P. (2013). Both resistance training and aerobic training reduce hepatic fat content in type 2 diabetic subjects with nonalcoholic fatty liver disease (the RAED2 randomized trial). Hepatology.

[38] Oh S., So R., Shida T., Matsuo T., Kim B., Akiyama K., Isobe T., Okamoto Y., Tanaka K., Shoda J. (2017). High-Intensity Aerobic Exercise Improves Both Hepatic Fat Content and Stiffness in Sedentary Obese Men with Nonalcoholic Fatty Liver Disease. Sci. Rep..

[39] Hashida R., Kawaguchi T., Bekki M., Omoto M., Matsuse H., Nago T., Takano Y., Ueno T., Koga H., George J. (2017). Aerobic vs. resistance exercise in non-alcoholic fatty liver disease: A systematic review. J. Hepatol..

[40] Zhang H.-J., Pan L.-L., Ma Z.-M., Chen Z., Huang Z.-F., Sun Q., Lu Y., Han C.-K., Lin M.-Z., Li X.-J. (2017). Long-term effect of exercise on improving fatty liver and cardiovascular risk factors in obese adults: A 1-year follow-up study. Diabetes Obes. Metab..

[41] Shen J., Wong G.L.-H., Chan H.L.-Y., Chan R.S.-M., Chan H.-Y., Chu W.C.-W., Cheung B.H.-K., Yeung D.K.-W., Li L.S., Sea M.M.-M. (2015). PNPLA3 gene polymorphism and response to lifestyle modification in patients with nonalcoholic fatty liver disease. J. Gastroenterol. Hepatol..

[42] Golabi P., Locklear C.T., Austin P., Afdhal S., Byrns M., Gerber L., Younossi Z.M. (2016). Effectiveness of exercise in hepatic fat mobilization in non-alcoholic fatty liver disease: Systematic review. World J. Gastroenterol..

[43] Thyfault J.P., Bergouignan A. (2020). Exercise and metabolic health: Beyond skeletal muscle. Diabetologia.

[44] Moreira J.B.N., Wohlwend M., Wisløff U. (2020). Exercise and cardiac health: Physiological and molecular insights. Nat. Metab..

[45] Nguyen N.T., Varela J.E. (2017). Bariatric surgery for obesity and metabolic disorders: State of the art. Nat. Rev. Gastroenterol. Hepatol..

[46] Lee Y., Doumouras A.G., Yu J., Brar K., Banfield L., Gmora S., Anvari M., Hong D. (2019). Complete Resolution of Nonalcoholic Fatty Liver Disease After Bariatric Surgery: A Systematic Review and Meta-analysis. Clin. Gastroenterol. Hepatol..

[47] Brunt E.M., Wong V.W.-S., Nobili V., Day C.P., Sookoian S., Maher J.J., Bugianesi E., Sirlin C.B., Neuschwander-Tetri B.A., Rinella M.E. (2015). Nonalcoholic fatty liver disease. Nat. Rev. Dis. Prim..

[48] Jiang X., Zheng J., Zhang S., Wang B., Wu C., Guo X. (2020). Advances in the Involvement of Gut Microbiota in Pathophysiology of NAFLD. Front. Med..

[49] Chu H., Duan Y., Yang L., Schnabl B. (2018). Small metabolites, possible big changes: A microbiota-centered view of non-alcoholic fatty liver disease. Gut.

[50] Charlton M.R., Burns J.M., Pedersen R.A., Watt K.D., Heimbach J.K., Dierkhising R.A. (2011). Frequency and Outcomes of Liver Transplantation for Nonalcoholic Steatohepatitis in the United States. Gastroenterology.

[51] Wang X., Li J., Riaz D., Shi G., Liu C., Dai Y. (2014). Outcomes of Liver Transplantation for Nonalcoholic Steatohepatitis: A Systematic Review and Meta-analysis. Clin. Gastroenterol. Hepatol..

[52] Burra P., Becchetti C., Germani G. (2020). NAFLD and liver transplantation: Disease burden, current management and future challenges. JHEP Rep..

[53] Gitto S., Vukotic R., Vitale G., Pirillo M., Villa E., Andreone P. (2016). Non-alcoholic steatohepatitis and liver transplantation. Dig. Liver Dis..

[54] Samji N.S., Heda R., Satapathy S.K. (2020). Peri-transplant management of nonalcoholic fatty liver disease in liver transplant candidates. Transl. Gastroenterol. Hepatol..

[55] Mikolasevic I., Filipec-Kanizaj T., Mijic M., Jakopcic I., Milic S., Hrstic I., Sobocan N., Stimac D., Burra P. (2018). Nonalcoholic fatty liver disease and liver transplantation—Where do we stand?. World J. Gastroenterol..

[56] Mantovani A., Byrne C., Scorletti E., Mantzoros C., Targher G. (2020). Efficacy and safety of anti-hyperglycaemic drugs in patients with non-alcoholic fatty liver disease with or without diabetes: An updated systematic review of randomized controlled trials. Diabetes Metab..

[57] LaVine J.E. (2011). Effect of Vitamin E or Metformin for Treatment of Nonalcoholic Fatty Liver Disease in Children and Adolescents. JAMA.

[58] Francque S., Szabo G., Abdelmalek M.F., Byrne C.D., Cusi K., Dufour J.-F., Roden M., Sacks F., Tacke F. (2021). Nonalcoholic steatohepatitis: The role of peroxisome proliferator-activated receptors. Nat. Rev. Gastroenterol. Hepatol..

[59] Sanyal A.J., Chalasani N., Kowdley K.V., McCullough A., Diehl A.M., Bass N.M., Neuschwander-Tetri B.A., LaVine J.E., Tonascia J., Unalp A. (2010). Pioglitazone, Vitamin E, or Placebo for Nonalcoholic Steatohepatitis. N. Engl. J. Med..

[60] Belfort R., Harrison S.A., Brown K., Darland C., Finch J., Hardies J., Balas B., Gastaldelli A., Tio F., Pulcini J. (2006). A Placebo-Controlled Trial of Pioglitazone in Subjects with Nonalcoholic Steatohepatitis. N. Engl. J. Med..

[61] Cusi K., Orsak B., Bril F., Lomonaco R., Hecht J., Ortiz-Lopez C., Tio F., Hardies J., Darland C., Musi N. (2016). Long-Term Pioglitazone Treatment for Patients With Nonalcoholic Steatohepatitis and Prediabetes or Type 2 Diabetes Mellitus. Ann. Intern. Med..

[62] Aithal G.P., Thomas J.A., Kaye P.V., Lawson A., Ryder S.D., Spendlove I., Austin A.S., Freeman J.G., Morgan L., Webber J. (2008). Randomized, Placebo-Controlled Trial of Pioglitazone in Nondiabetic Subjects with Nonalcoholic Steatohepatitis. Gastroenterology.

[63] Ratziu V., Giral P., Jacqueminet S., Charlotte F., Hartemann–Heurtier A., Serfaty L., Podevin P., Lacorte J., Bernhardt C., Bruckert E. (2008). Rosiglitazone for Nonalcoholic Steatohepatitis: One-Year Results of the Randomized Placebo-Controlled Fatty Liver Improvement With Rosiglitazone Therapy (FLIRT) Trial. Gastroenterology.

[64] Musso G., Cassader M., Paschetta E., Gambino R. (2017). Thiazolidinediones and Advanced Liver Fibrosis in Nonalcoholic Steatohepatitis. JAMA Intern. Med..

[65] Ratziu V., Harrison S.A., Francque S., Bedossa P., Lehert P., Serfaty L., Romero-Gomez M., Boursier J., Abdelmalek M., Caldwell S. (2016). Elafibranor, an Agonist of the Peroxisome Proliferator–Activated Receptor–α and –δ, Induces Resolution of Nonalcoholic Steatohepatitis Without Fibrosis Worsening. Gastroenterology.

[66] Mantovani A., Petracca G., Beatrice G., Csermely A., Lonardo A., Targher G. (2021). Glucagon-Like Peptide-1 Receptor Agonists for Treatment of Nonalcoholic Fatty Liver Disease and Nonalcoholic Steatohepatitis: An Updated Meta-Analysis of Randomized Controlled Trials. Metabolism.

[67] Armstrong M.J., Houlihan D.D., Rowe I.A., Clausen W.H.O., Elbrønd B., Gough S.C.L., Tomlinson J.W., Newsome P.N. (2012). Safety and efficacy of liraglutide in patients with type 2 diabetes and elevated liver enzymes: Individual patient data meta-analysis of the LEAD program. Aliment. Pharmacol. Ther..

[68] Armstrong M.J., Gaunt P., Aithal G.P., Barton D., Hull D., Parker R., Hazlehurst J.M., Guo K., Abouda G., Aldersley M.A. (2016). Liraglutide safety and efficacy in patients with non-alcoholic steatohepatitis (LEAN): A multicentre, double-blind, randomised, placebo-controlled phase 2 study. Lancet.

[69] Harrison S.A., Calanna S., Cusi K., Linder M., Okanoue T., Ratziu V., Sanyal A., Sejling A.-S., Newsome P.N. (2020). Semaglutide for the treatment of non-alcoholic steatohepatitis: Trial design and comparison of non-invasive biomarkers. Contemp. Clin. Trials.

[70] Kuchay M.S., Krishan S., Mishra S.K., Choudhary N.S., Singh M.K., Wasir J.S., Kaur P., Gill H.K., Bano T., Farooqui K.J. (2020). Effect of dulaglutide on liver fat in patients with type 2 diabetes and NAFLD: Randomised controlled trial (D-LIFT trial). Diabetologia.

[71] Newsome P.N., Buchholtz K., Cusi K., Linder M., Okanoue T., Ratziu V., Sanyal A.J., Sejling A.-S., Harrison S.A. (2020). A Placebo-Controlled Trial of Subcutaneous Semaglutide in Nonalcoholic Steatohepatitis. N. Engl. J. Med..

[72] Mantovani A., Petracca G., Csermely A., Beatrice G., Targher G. (2020). Sodium-Glucose Cotransporter-2 Inhibitors for Treatment of Nonalcoholic Fatty Liver Disease: A Meta-Analysis of Randomized Controlled Trials. Metabolism.

[73] Coelho F.D.S., Borges-Canha M., Von Hafe M., Neves J.S., Vale C., Leite A.R., Carvalho D., Moreira A.L. (2020). Effects of SGLT2 inhibitors on liver parameters and steatosis: A meta-analysis of randomized clinical trials. Diabetes/Metab. Res. Rev..

[74] Simon T.G., Henson J., Osganian S., Masia R., Chan A.T., Chung R.T., Corey K.E. (2019). Daily Aspirin Use Associated with Reduced Risk for Fibrosis Progression in Patients with Nonalcoholic Fatty Liver Disease. Clin. Gastroenterol. Hepatol..

[75] Malehmir M., Pfister D., Gallage S., Szydlowska M., Inverso D., Kotsiliti E., Leone V., Peiseler M., Surewaard B.G.J., Rath D. (2019). Platelet GPIbα is a mediator and potential interventional target for NASH and subsequent liver cancer. Nat. Med..

[76] Neuschwander-Tetri B.A., Loomba R., Sanyal A.J., Lavine J.E., Van Natta M.L., Abdelmalek M.F., Chalasani N., Dasarathy S., Diehl A.M., Hameed B. (2015). Farnesoid X nuclear receptor ligand obeticholic acid for non-cirrhotic, non-alcoholic steatohepatitis (FLINT): A multicentre, randomised, placebo-controlled trial. Lancet.

[77] Younossi Z.M., Ratziu V., Loomba R., Rinella M., Anstee Q.M., Goodman Z., Bedossa P., Geier A., Beckebaum S., Newsome P.N. (2019). Obeticholic acid for the treatment of non-alcoholic steatohepatitis: Interim analysis from a multicentre, randomised, placebo-controlled phase 3 trial. Lancet.

[78] Loomba R., Morgan E., Watts L., Xia S., Hannan L.A., Geary R.S., Baker B.F., Bhanot S. (2020). Novel antisense inhibition of diacylglycerol O-acyltransferase 2 for treatment of non-alcoholic fatty liver disease: A multicentre, double-blind, randomised, placebo-controlled phase 2 trial. Lancet Gastroenterol. Hepatol..

[79] Harrison S.A., Bashir M.R., Guy C.D., Zhou R., Moylan C.A., Frias J.P., Alkhouri N., Bansal M.B., Baum S., Neuwschwander-Tetri B.A. (2019). Resmetirom (MGL-3196) for the treatment of non-alcoholic steatohepatitis: A multicentre, randomised, double-blind, placebo-controlled, phase 2 trial. Lancet.

[80] Friedman S.L., Ratziu V., Harrison S.A., Abdelmalek M.F., Aithal G.P., Caballeria J., Francque S., Farrell G., Kowdley K.V., Craxi A. (2018). A randomized, placebo-controlled trial of cenicriviroc for treatment of nonalcoholic steatohepatitis with fibrosis. Hepatology.

[81] Harrison S.A., Goodman Z., Jabbar A., Vemulapalli R., Younes Z.H., Freilich B., Sheikh M.Y., Schattenberg J.M., Kayali Z., Zivony A. (2020). A randomized, placebo-controlled trial of emricasan in patients with NASH and F1-F3 fibrosis. J. Hepatol..

[82] Harrison S.A., Abdelmalek M.F., Caldwell S., Shiffman M.L., Diehl A.M., Ghalib R., Lawitz E.J., Rockey D.C., Schall R.A., Jia C. (2018). Simtuzumab Is Ineffective for Patients with Bridging Fibrosis or Compensated Cirrhosis Caused by Nonalcoholic Steatohepatitis. Gastroenterology.

[83] Harrison S.A., Wong V.W.-S., Okanoue T., Bzowej N., Vuppalanchi R., Younes Z., Kohli A., Sarin S., Caldwell S.H., Alkhouri N. (2020). Selonsertib for patients with bridging fibrosis or compensated cirrhosis due to NASH: Results from randomized phase III STELLAR trials. J. Hepatol..

[84] American Diabetes Association (2019). 9. Pharmacologic Approaches to Glycemic Treatment: Standards of Medical Care in Diabetes—2019. Diabetes Care.

[85] Raschi E., Mazzotti A., Poluzzi E., De Ponti F., Marchesini G. (2018). Pharmacotherapy of type 2 diabetes in patients with chronic liver disease: Focus on nonalcoholic fatty liver disease. Expert Opin. Pharmacother..

[86] Foretz M., Guigas B., Bertrand L., Pollak M., Viollet B. (2014). Metformin: From Mechanisms of Action to Therapies. Cell Metab..

[87] Foretz M., Guigas B., Viollet B. (2019). Understanding the glucoregulatory mechanisms of metformin in type 2 diabetes mellitus. Nat. Rev. Endocrinol..

[88] Kaplan D.E., Serper M., John B.V., Tessiatore K.M., Lerer R., Mehta R., Fox R., Aytaman A., Baytarian M., Hunt K. (2020). Effects of Metformin Exposure on Survival in a Large National Cohort of Patients with Diabetes and Cirrhosis. Clin. Gastroenterol. Hepatol..

[89] Vilar-Gomez E., Vuppalanchi R., Desai A.P., Gawrieh S., Ghabril M., Saxena R., Cummings O.W., Chalasani N. (2019). Long-term metformin use may improve clinical outcomes in diabetic patients with non-alcoholic steatohepatitis and bridging fibrosis or compensated cirrhosis. Aliment. Pharmacol. Ther..

[90] Zhou J., Ke Y., Lei X., Wu T., Li Y., Bao T., Tang H., Zhang C., Wu X., Wang G. (2020). Meta-analysis: The efficacy of metformin and other anti-hyperglycemic agents in prolonging the survival of hepatocellular carcinoma patients with type 2 diabetes. Ann. Hepatol..

[91] Schulte L., Scheiner B., Voigtländer T., Koch S., Schweitzer N., Marhenke S., Ivanyi P., Manns M.P., Rodt T., Hinrichs J.B. (2019). Treatment with metformin is associated with a prolonged survival in patients with hepatocellular carcinoma. Liver Int..

[92] Singh S., Singh P.P., Singh A.G., Murad M.H., Sanchez W. (2013). Anti-Diabetic Medications and the Risk of Hepatocellular Cancer: A Systematic Review and Meta-Analysis. Am. J. Gastroenterol..

[93] Chen Y.C., Li H., Wang J. (2020). Mechanisms of metformin inhibiting cancer invasion and migration. Am. J. Transl. Res..

[94] Dormandy J.A., Charbonnel B., Eckland D.J.A., Erdmann E., Massi-Benedetti M., Moules I.K., Skene A.M., Tan M.H., Lefèbvre P.J., Murray G.D. (2005). Secondary prevention of macrovascular events in patients with type 2 diabetes in the PROactive Study (PROspective pioglitAzone Clinical Trial In macroVascular Events): A randomised controlled trial. Lancet.

[95] Spence J.D., Viscoli C.M., Inzucchi S.E., Dearborn-Tomazos J., Ford G.A., Gorman M., Furie K.L., Lovejoy A.M., Young L.H., Kernan W.N. (2019). Pioglitazone Therapy in Patients with Stroke and Prediabetes. JAMA Neurol..

[96] Kawaguchi-Suzuki M., Cusi K., Bril F., Gong Y., Langaee T., Frye R.F. (2018). A Genetic Score Associates with Pioglitazone Response in Patients with Non-alcoholic Steatohepatitis. Front. Pharmacol..

[97] Friedman S.L., Neuschwander-Tetri B.A., Rinella M., Sanyal A.J. (2018). Mechanisms of NAFLD development and therapeutic strategies. Nat. Med..

[98] Kaul U., Parmar D., Manjunath K., Shah M., Parmar K., Patil K.P., Jaiswal A. (2019). MNew dual peroxisome proliferator activated receptor agonist—Saroglitazar in diabetic dyslipidemia and non-alcoholic fatty liver disease: Integrated analysis of the real world evidence. Cardiovasc. Diabetol..

[99] Kalavalapalli S., Bril F., Guingab J., Vergara A., Garrett T.J., Sunny N.E., Cusi K. (2019). Impact of exenatide on mitochondrial lipid metabolism in mice with nonalcoholic steatohepatitis. J. Endocrinol..

[100] Marso S.P., Daniels G.H., Brown-Frandsen K., Kristensen P., Mann J.F., Nauck M.A., Nissen S.E., Pocock S., Poulter N.R., Ravn L.S. (2016). Liraglutide and Cardiovascular Outcomes in Type 2 Diabetes. N. Engl. J. Med..

[101] Kristensen S.L., Rørth R., Jhund P.S., Docherty K.F., Sattar N., Preiss D., Køber L., Petrie M.C., McMurray J.J. (2019). Cardiovascular, mortality, and kidney outcomes with GLP-1 receptor agonists in patients with type 2 diabetes: A systematic review and meta-analysis of cardiovascular outcome trials. Lancet Diabetes Endocrinol..

[102] Katsiki N., Perakakis N., Mantzoros C. (2019). Effects of sodium-glucose co-transporter-2 (SGLT2) inhibitors on non-alcoholic fatty liver disease/non-alcoholic steatohepatitis: Ex quo et quo vadimus?. Metabolism.

[103] Neuen B.L., Young T., Heerspink H.J.L., Neal B., Perkovic V., Billot L., Mahaffey K.W., Charytan D.M., Wheeler D.C., Arnott C. (2019). SGLT2 inhibitors for the prevention of kidney failure in patients with type 2 diabetes: A systematic review and meta-analysis. Lancet Diabetes Endocrinol..

[104] Zelniker T.A., Wiviott S.D., Raz I., Im K., Goodrich E.L., Bonaca M.P., Mosenzon O., Kato E.T., Cahn A., Furtado R.H.M. (2019). SGLT2 inhibitors for primary and secondary prevention of cardiovascular and renal outcomes in type 2 diabetes: A systematic review and meta-analysis of cardiovascular outcome trials. Lancet.

[105] Pose E., Trebicka J., Mookerjee R.P., Angeli P., Ginès P. (2019). Statins: Old drugs as new therapy for liver diseases?. J. Hepatol..

[106] Ekstedt M., Franzèn L.E., Mathiesen U.L., Holmqvist M., Bodemar G., Kechagias S. (2007). Statins in non-alcoholic fatty liver disease and chronically elevated liver enzymes: A histopathological follow-up study. J. Hepatol..

[107] Cohen D.E., Anania F.A., Chalasani N. (2006). An Assessment of Statin Safety by Hepatologists. Am. J. Cardiol..

[108] Athyros V.G., Tziomalos K., Gossios T.D., Griva T., Anagnostis P., Kargiotis K., Pagourelias E.D., Theocharidou E., Karagiannis A., Mikhailidis D.P. (2010). Safety and efficacy of long-term statin treatment for cardiovascular events in patients with coronary heart disease and abnormal liver tests in the Greek Atorvastatin and Coronary Heart Disease Evaluation (GREACE) Study: A post-hoc analysis. Lancet.

[109] Dongiovanni P., Petta S., Mannisto V., Mancina R.M., Pipitone R.M., Karja V., Maggioni M., Kakela P., Wiklund O., Mozzi E. (2015). Statin use and non-alcoholic steatohepatitis in at risk individuals. J. Hepatol..

[110] Lee J.I., Lee H.W., Lee K.S., Lee H.S., Park J.-Y. (2021). Effects of Statin Use on the Development and Progression of Nonalcoholic Fatty Liver Disease: A Nationwide Nested Case-Control Study. Am. J. Gastroenterol..

[111] Nascimbeni F., Aron-Wisnewsky J., Pais R., Tordjman J., Poitou C., Charlotte F., Bedossa P., Poynard T., Clément K., Ratziu V. (2016). Statins, antidiabetic medications and liver histology in patients with diabetes with non-alcoholic fatty liver disease. BMJ Open Gastroenterol..

[112] Athyros V.G., Alexandrides T.K., Bilianou H., Cholongitas E., Doumas M., Ganotakis E.S., Goudevenos J., Elisaf M.S., Germanidis G., Giouleme O. (2017). The use of statins alone, or in combination with pioglitazone and other drugs, for the treatment of non-alcoholic fatty liver disease/non-alcoholic steatohepatitis and related cardiovascular risk. An Expert Panel Statement. Metabolism.

[113] Nakade Y., Murotani K., Inoue T., Kobayashi Y., Yamamoto T., Ishii N., Ohashi T., Ito K., Fukuzawa Y., Yoneda M. (2017). Ezetimibe for the treatment of non-alcoholic fatty liver disease: A meta-analysis. Hepatol. Res..

[114] Dewidar B., Kahl S., Pafili K., Roden M. (2020). Metabolic liver disease in diabetes—From mechanisms to clinical trials. Metabolism.

[115] Lee C.-H., Fu Y., Yang S.-J., Chi C.-C. (2020). Effects of Omega-3 Polyunsaturated Fatty Acid Supplementation on Non-Alcoholic Fatty Liver: A Systematic Review and Meta-Analysis. Nutrients.

[116] Valenzuela R., Videla L.A. (2020). Impact of the Co-Administration of N-3 Fatty Acids and Olive Oil Components in Preclinical Nonalcoholic Fatty Liver Disease Models: A Mechanistic View. Nutrients.

[117] Parker H.M., Johnson N.A., Burdon C.A., Cohn J.S., O’Connor H.T., George J. (2012). Omega-3 supplementation and non-alcoholic fatty liver disease: A systematic review and meta-analysis. J. Hepatol..

[118] Wargny M., Ducluzeau P.-H., Petit J.-M., Le May C., Smati S., Arnaud L., Pichelin M., Bouillet B., Lannes A., Blanchet O. (2018). Circulating PCSK9 levels are not associated with the severity of hepatic steatosis and NASH in a high-risk population. Atherosclerosis.

[119] Theocharidou E., Papademetriou M., Reklou A., Sachinidis A., Boutari C., Giouleme O. (2019). The Role of PCSK9 in the Pathogenesis of Non-alcoholic Fatty Liver Disease and the Effect of PCSK9 Inhibitors. Curr. Pharm. Des..

[120] Jonsson J.R., Clouston A.D., Ando Y., Kelemen L.I., Horn M.J., Adamson M.D., Purdie D.M., Powell E.E. (2001). Angiotensin-Converting Enzyme Inhibition Attenuates the Progression of Rat Hepatic Fibrosis. Gastroenterology.

[121] Moreno M., Gonzalo T., Kok R.J., Sancho-Bru P., Van Beuge M., Swart J., Prakash J., Temming K., Fondevila C., Beljaars L. (2010). Reduction of advanced liver fibrosis by short-term targeted delivery of an angiotensin receptor blocker to hepatic stellate cells in rats. Hepatology.

[122] Hirose A., Ono M., Saibara T., Nozaki Y., Masuda K., Yoshioka A., Takahashi M., Akisawa N., Iwasaki S., Oben J.A. (2007). Angiotensin II type 1 receptor blocker inhibits fibrosis in rat nonalcoholic steatohepatitis. Hepatology.

[123] Park J.G., Mok J.S., Han Y.I., Park T.S., Kang K.W., Choi C.S., Park H.D., Park J. (2019). Connectivity mapping of angiotensin-PPAR interactions involved in the amelioration of non-alcoholic steatohepatitis by Telmisartan. Sci. Rep..

[124] Yokohama S., Yoneda M., Haneda M., Okamoto S., Okada M., Aso K., Hasegawa T., Tokusashi Y., Miyokawa N., Nakamura K. (2004). Therapeutic efficacy of an angiotensin II receptor antagonist in patients with nonalcoholic steatohepatitis. Hepatology.

[125] Georgescu E.F., Ionescu R., Niculescu M., Mogoanta L., Vancica L. (2009). Angiotensin-receptor blockers as therapy for mild-to-moderate hypertension-associated non-alcoholic steatohepatitis. World J. Gastroenterol..

[126] Torres D.M., Jones F.J., Shaw J.C., Williams C.D., Ward J.A., Harrison S.A. (2011). Rosiglitazone versus rosiglitazone and metformin versus rosiglitazone and losartan in the treatment of nonalcoholic steatohepatitis in humans: A 12-month randomized, prospective, open-label trial. Hepatology.

[127] McPherson S., Wilkinson N., Tiniakos D., Wilkinson J., Burt A.D., McColl E., Stocken D.D., Steen N., Barnes J., Goudie N. (2017). A randomised controlled trial of losartan as an anti-fibrotic agent in non-alcoholic steatohepatitis. PLoS ONE.

[128] He W., Xu Y., Ren X., Xiang D., Lei K., Zhang C., Liu D. (2019). Vitamin E Ameliorates Lipid Metabolism in Mice with Nonalcoholic Fatty Liver Disease via Nrf2/CES1 Signaling Pathway. Dig. Dis. Sci..

[129] Uchida D., Takaki A., Adachi T., Okada H. (2018). Beneficial and Paradoxical Roles of Anti-Oxidative Nutritional Support for Non-Alcoholic Fatty Liver Disease. Nutrients.

[130] Kong B., Luyendyk J.P., Tawfik O., Guo G.L. (2008). Farnesoid X Receptor Deficiency Induces Nonalcoholic Steatohepatitis in Low-Density Lipoprotein Receptor-Knockout Mice Fed a High-Fat Diet. J. Pharmacol. Exp. Ther..

[131] Siddiqui M.S., Van Natta M.L., Connelly M.A., Vuppalanchi R., Neuschwander-Tetri B.A., Tonascia J., Guy C., Loomba R., Dasarathy S., Wattacheril J. (2020). Impact of obeticholic acid on the lipoprotein profile in patients with non-alcoholic steatohepatitis. J. Hepatol..

[132] Pockros P.J., Fuchs M., Freilich B., Schiff E., Kohli A., Lawitz E.J., Hellstern P.A., Owens-Grillo J., Van Biene C., Shringarpure R. (2019). CONTROL: A randomized phase 2 study of obeticholic acid and atorvastatin on lipoproteins in nonalcoholic steatohepatitis patients. Liver Int..

[133] Gawrieh S., Guo X., Tan J., Lauzon M., Taylor K.D., Loomba R., Cummings O.W., Pillai S., Bhatnagar P., Kowdley K.V. (2019). A Pilot Genome-Wide Analysis Study Identifies Loci Associated with Response to Obeticholic Acid in Patients With NASH. Hepatol. Commun..

[134] Harrison S.A., Rinella E.M., Abdelmalek M.F., Trotter J.F., Paredes A.H., Arnold H.L., Kugelmas M., Bashir M.R., Jaros M.J., Ling L. (2018). NGM282 for treatment of non-alcoholic steatohepatitis: A multicentre, randomised, double-blind, placebo-controlled, phase 2 trial. Lancet.

[135] Harrison S.A., Rossi S.J., Paredes A.H., Trotter J.F., Bashir M.R., Guy C.D., Banerjee R., Jaros M.J., Owers S., Baxter B.A. (2020). NGM282 Improves Liver Fibrosis and Histology in 12 Weeks in Patients with Nonalcoholic Steatohepatitis. Hepatology.

[136] Stone S.J., Levin M.C., Zhou P., Han J., Walther T.C., Farese R.V. (2009). The Endoplasmic Reticulum Enzyme DGAT2 Is Found in Mitochondria-associated Membranes and Has a Mitochondrial Targeting Signal That Promotes Its Association with Mitochondria. J. Biol. Chem..

[137] Krenkel O., Puengel T., Govaere O., Abdallah A.T., Mossanen J.C., Kohlhepp M., Liepelt A., Lefebvre E., Luedde T., Hellerbrand C. (2018). Therapeutic inhibition of inflammatory monocyte recruitment reduces steatohepatitis and liver fibrosis. Hepatology.

[138] Rotman Y., Sanyal A.J. (2017). Current and upcoming pharmacotherapy for non-alcoholic fatty liver disease. Gut.

[139] Barreyro F.J., Holod S., Finocchietto P.V., Camino A.M., Aquino J.B., Avagnina A., Carreras M.C., Poderoso J.J., Gores G.J. (2015). The pan-caspase inhibitor Emricasan (IDN-6556) decreases liver injury and fibrosis in a murine model of non-alcoholic steatohepatitis. Liver Int..

[140] Rinella M.E., Noureddin M. (2020). STELLAR 3 and STELLAR 4: Lessons from the fall of Icarus. J. Hepatol..

[141] Singh S., Khera R., Allen A.M., Murad M.H., Loomba R. (2015). Comparative effectiveness of pharmacological interventions for nonalcoholic steatohepatitis: A systematic review and network meta-analysis. Hepatology.

[142] Suk K.T., Kim D.J. (2019). Gut microbiota: Novel therapeutic target for nonalcoholic fatty liver disease. Expert Rev. Gastroenterol. Hepatol..

[143] Koopman N., Molinaro A., Nieuwdorp M., Holleboom A.G. (2019). Review article: Can bugs be drugs? The potential of probiotics and prebiotics as treatment for non-alcoholic fatty liver disease. Aliment. Pharmacol. Ther..

[144] Scorletti E., Afolabi P.R., Miles E.A., Smith D.E., Almehmadi A., AlShathry A., Childs C.E., Del Fabbro S., Bilson J., Moyses H.E. (2020). Synbiotics Alter Fecal Microbiomes, but Not Liver Fat or Fibrosis, in a Randomized Trial of Patients with Nonalcoholic Fatty Liver Disease. Gastroenterology.

[145] Dufour J.-F., Caussy C., Loomba R. (2020). Combination therapy for non-alcoholic steatohepatitis: Rationale, opportunities and challenges. Gut.

[146] Harrison S.A., Ratziu V., Boursier J., Francque S., Bedossa P., Majd Z., Cordonnier G., Ben Sudrik F., Darteil R., Liebe R. (2020). A blood-based biomarker panel (NIS4) for non-invasive diagnosis of non-alcoholic steatohepatitis and liver fibrosis: A prospective derivation and global validation study. Lancet Gastroenterol. Hepatol..

